# Towards high atom economy in whole-cell redox biocatalysis: up-scaling light-driven cyanobacterial ene-reductions in a flat panel photobioreactor†

**DOI:** 10.1039/d4gc05686h

**Published:** 2025-01-14

**Authors:** Hanna C. Grimm, Peter Erlsbacher, Hitesh Medipally, Lenny Malihan-Yap, Lucija Sovic, Johannes Zöhrer, Sergey N. Kosourov, Yagut Allahverdiyeva, Caroline E. Paul, Robert Kourist

**Affiliations:** a Institute for Molecular Biotechnology TU Graz Petersgasse 14/1 A-8010 Graz Austria hanna.grimm@iftc.uni-hannover.de kourist@tugraz.at; b Molecular Plant Biology, Department of Life Technologies, University of Turku 20014 Turku Finland; c Department of Biotechnology, Delft University of Technology Van der Maasweg 9 2629 HZ Delft The Netherlands; d acib GmbH Krenngasse 37 8010 Graz Austria

## Abstract

Light-driven biotransformations in recombinant cyanobacteria benefit from the atom-efficient regeneration of reaction equivalents like NADPH from water and light by oxygenic photosynthesis. The self-shading of photosynthetic cells throughout the reaction volume, along with the need for extended light paths, limits adequate light supply and significantly restricts the potential for upscaling. Here, we present a flat panel photobioreactor (1 cm optical path length) as a scalable system to provide efficient illumination at high cell densities. The genes of five ene-reductases from different classes were expressed in *Synechocystis* sp. PCC 6803. The strains were characterised in the light-driven reduction of a set of prochiral substrates. With specific activities up to 150 U g_CDW_^−1^ under standard conditions in small-scale reactions, the recombinant strains harbouring the ene-reductases TsOYE C25G I67T and OYE3 showed the highest specific activities observed so far in photobiotransformations and were selected for the up-scale in the flat panel photobioreactor in 120 mL-scale. The strain producing OYE3 exhibited a specific activity as high as 56.1 U g_CDW_^−1^. The corresponding volumetric productivity of 1 g L^−1^ h^−1^ compares favourably to other photosynthesis-driven processes. This setup facilitated the conversion of 50 mM over approximately 8 hours to an isolated yield of 87%. The atom economy of 88% compares favourably to the use of the sacrificial co-substrates glucose and formic acid with 49% and 78%, respectively. Determination of the complete *E*-Factor of 203 including water reveals that the volumetric yield and water required for cultivation are crucial for the sustainability. In summary, our results point out key factors for the sustainability of light-driven whole-cell biotransformations, and provide a solid basis for future optimisation and up-scale campaigns of photosynthesis-driven bioproduction.

## Abstract 2

Light-driven biotransformations in recombinant cyanobacteria benefit from the atom-efficient regeneration of reaction equivalents like NADPH from water and light by oxygenic photosynthesis.

## Graphical abstract

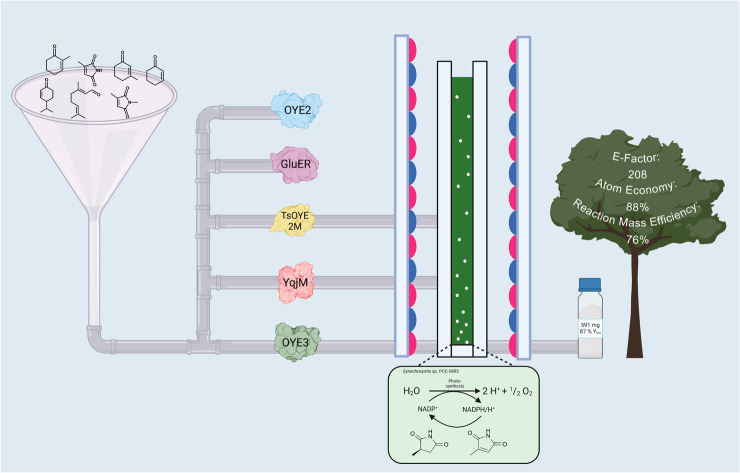

**Green foundation**

1. Our work demonstrates that cyanobacterial photobiotransformations can substitute glucose as electron donor for redox biocatalysis, and with very high reaction rates.

2. A process conducted in a scalable flat-panel photobioreactor shows significantly improved atom economy and a reaction mass efficiency comparable to heterotrophic microorganisms. While the high volumetric productivity stands out, our analysis indicate the relatively low substrate concentration, the water demand of the cultivation and the electricity required for illumination as key factors for sustainability.

3. Future work will focus on achieving higher substrate concentrations and optimizing conditions of the cultivation, aiming to provide a solid basis for a life-cycle analysis to provide quantitative evidence of the sustainability advantages of photosynthesis-driven redox biocatalysis.

## Introduction

Ene-reductases (ERs) catalyse the reduction of activated C=C-double bonds under ambient conditions.^1,2^ They contain a non-covalently bound flavin mononucleotide cofactor (FMN) which is reduced to FMNH_2_ in the first, reductive step of the reaction. For this, ERs accept both nicotinamide cofactors NADH and NADPH as hydride donor but usually with a strong preference for the phosphorylated form.^3–5^ In the second, oxidative step, the hydride is further transferred to the C_β_-atom of the C=C bond within the substrate while FMNH_2_ is re-oxidised. The stoichiometric demand for NAD(P)H requires a recycling system to make ER-catalysed reactions economically feasible. The most common strategies involve a second enzymatic step that oxidises a sacrificial co-substrate and simultaneously reduces NAD(P)^+^. Co-substrates such as glucose reduce the atom economy of a process, since only few electrons of the molecules are dedicated for cofactor recycling.^6^ In addition, oxidised electron donors and biomass formed from the sugars are undesired side-products which must be separated in downstream processing requiring energy and effort.^7^ Photosynthetic organisms like cyanobacteria harness light for the regeneration of NADPH and ATP. This process is initiated with light-dependent water oxidation by photosystem II, and the released electrons are shuttled *via* cytochrome *b*_6_*f* complex towards photosystem I. This complex facilitates the light-driven reduction of ferredoxin, which then transfers electrons to NADP^+^ reductase for regenerating NADPH.^8^ Simultaneously, ATP is regenerated by a proton motive force established across the thylakoid membrane due to photosynthetic electron transfer.^9^

The ability of oxygenic photosynthesis allows cyanobacteria to provide NADPH and O_2_ for recombinant redox reactions.^10,11^ So far, several oxidoreductases like imine reductases,^12^ monooxygenases^13–19^ and dehydrogenases^20,21^ have been recombinantly produced in cyanobacteria and the activity of the strains was investigated. Among them, the light-driven ene-reduction resulted in the highest specific activities. In 2016, Köninger *et al.*^22^ recombinantly produced the ene-reductase YqjM from *Bacillus subtilis* in the cyanobacterium *Synechocystis* sp. PCC 6803 (henceforth *Synechocystis*) and achieved high specific activities (>123 U g_CDW_^−1^). In 2020, we showed that the reduction of 2-methylmaleimide was limited by NADPH availability. Detailed kinetic investigation proved the oxidation of the cofactor as the rate limiting factor.^5^d-Glucose addition during photobiotransformations resulted in mixotrophic conditions, which increased activity due to the regeneration of NAD(P)H by glycolytic pathways.^23^ All these studies indicate that YqjM operates under its *K*_D_ for NADPH which is further supported by a recent study reporting nanomolar concentrations for NADPH in *Synechocystis*.^24^ It should be noted that in such a situation, both a higher concentration of enzyme or an enzyme with a higher specific activity increases the reaction rate. To identify enzymes with potentially higher whole-cell activity in *Synechocystis* (resulting from either better functional production or from higher activity), we investigated a set of ERs from different classes.

According to an analysis by Böhmer *et al.*,^14^ ERs can be classified into six classes that show differences in the origin, substrate scope and oligomeric state. Class I and II enzymes occur primarily as monomers or dimers and differ mainly in their origin and substrate preferences. For instance, class II enzymes originate exclusively from fungi. In contrast, class III enzymes occur in tetramers or higher oligomeric states and tend to be more tolerant towards cosolvents and temperature.^2^ Classes IV–VI, however, remain less explored, with only a limited number of members characterized to date. Sequence alignment confirmed that members of classes IV–VI share characteristic motifs from both class I/II and class III.^2,25^ We chose members of the three best-characterized classes (I–III) where we expected the highest likelihood of finding a highly active enzyme suitable for our approach. Furthermore, selected candidates should be known for an alternative substrate preference or otherwise for an opposite or improved selectivity. With YqjM, we already had a good class III candidate at hand.^26^ From class Ic, GluER from *Gluconobacter oxydans* was chosen which was well described by Richter *et al.* in 2011.^27^ This enzyme was shown to exhibit excellent selectivity and activity on substrates like ketoisophorone (product ee >99% (*R*)) and citral (product ee >99% (*S*)). ERs from class II like the two well-characterised OYE2 and OYE3 from *Saccharomyces cerevisiae* are known to be more active on cyclic enones.^28^ In general, most ERs among all classes exhibit similar enantioselectivity and stereocomplementary pairs are rare. For example, all selected ERs are (*R*)-selective when converting 2-methylcyclohexenone or maleimides like 2-methylmaleimide and 2-methyl-*N*-methylmaleimide. However, OYE2 and OYE3 show a preferential reduction of *E*-citral to form (*R*)-citronellal over *Z*-citral leading to (*S*)-citronellal, whereas GluER converts both *E*- and *Z*-isomers to (*S*)-citronellal.^29^ We decided to include the variant C25G I67T^30^ of the class III TsOYE (TsOYE_2M) from *Thermus scotoductus* in the screening, which was reported to have similar conversions^30,31^ as the wild type enzyme. Compared to the other ene-reductases, it provides opposite selectivity on 2-methylcyclohexenone (product ee = 14% (*S*)) and (*S*)-carvone (ee = 88% (2*S*,5*S*)).^31^ Additionally, it accepts β-substituted substrates such as 3-methylcyclohexenone, with opposite selectivity to that of OYE2.^31^

**Fig. 1:**
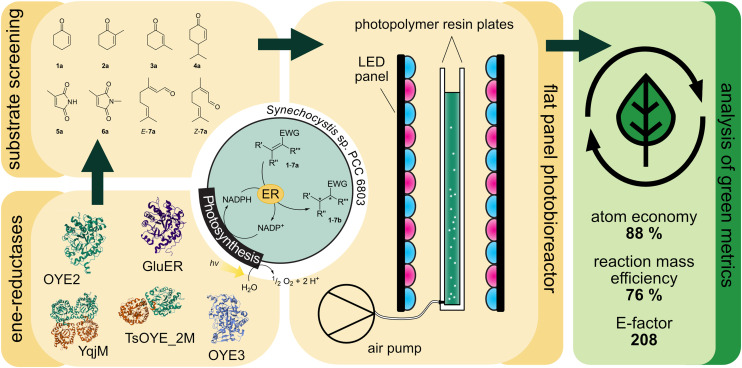
Light-driven asymmetric alkene reductions in recombinant *Synechocystis* sp. PCC 6803. Ene-reductases from different classes are produced in the cyanobacterium and the substrate scope of the strains was investigated. The best producing strains were investigated in the flat panel photobioreactor and the green metrics are analysed.

Due to NADPH limitation of the process, ensuring adequate light supply is crucial for industrial applications. While illuminating small volumes is relatively straightforward, up-scaling of photobioreactors for efficient light distribution poses a significant challenge. Up-scaling of photobiotransformations in different reactor types such as a stirred tank reactor (STR)^17,32^ and a bubble column reactor (BCR) with internal LED-illumination^33^ clearly indicated the self-shading of the cells as a crucial limitation. Capillary reactors^34,35^ showed higher production rates due to the high light penetration and the high surface area to volume ratio. However, scaling-up needs to be accomplished by parallelising reactor setups since larger diameters diminish the effect of efficient irradiation.^36^ Besides light availability, oxygen accumulation can cause photodamage in closed systems and the limited supply of CO_2_ might present additional obstacles. Flat panel photobioreactors (FPBR) are widely used for the cultivation of photoautotrophic microorganisms.^37^ They can be applied with large volumes and provide an efficient geometry for external illumination, which can be easily increased in volume with a low trade-off in light availability. This system presented itself as a practical and scalable option for up-scale of photobiotransformations (Fig. 1). Finally, we aim to analyse the sustainability of our process by calculating atom economies and *E*-factors.

## Results and discussion

### Fine-tuning expression by rhamnose-inducible promoter

A tight control of the protein production would be advantageous for enhancing reaction speed for biotransformations and their scale-up reactions. For this purpose, we first aimed to use the rhamnose-inducible promoter system which was previously characterised to produce the enhanced yellow fluorescent enzyme (eYFP) in *Synechocystis*.^38^ While we were able to replicate the production of eYFP in *Synechocystis* (Fig. S1†), we could not detect any activity in *Synechocystis* strains harbouring YqjM under the control of the rhamnose-promoter, neither on the self-replicating plasmid nor after genome integration. SDS-PAGE analysis revealed that no protein was produced upon rhamnose induction while eYFP production was clearly visible (Fig. S3†). Therefore, we focused on the well-established strong P_*cpc*_ promoter for further work on the ERs.

### Substrate scope and activity of the strains

**Fig. 2:**
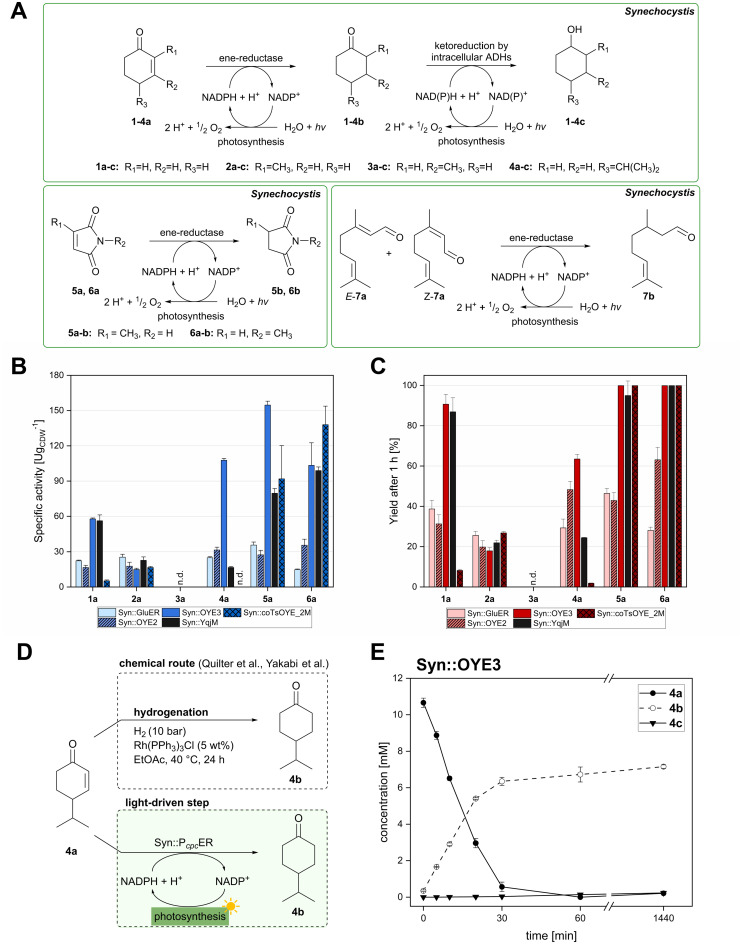
Substrate screening with *Synechocystis* harbouring different ene-reductases. (A) Reaction equations showing all possible products including side product of the interfering ketoreduction. (B) Specific activity and (C) product formation after 1 h. 7a was excluded from both graphs because no activity could be calculated, and no product was detected for any of the strains after 1 h. (D) Chemical reduction^40,41^ and our proposed biocatalytic route to reduce 4a into the ketone 4b and (E) reaction progress for the conversion of 4a by Syn::P_*cpc*_OYE3. Reaction conditions for all biotransformations: *c*(*S*) = 10 mM, cell density = 2.4 g_CDW_ L^−1^, 150 μmol_photons_ m^−2^ s^−1^, *V* = 1 mL, n.d. = not detected; results include data of at least three biological replicates.

The light-driven reduction of C=C double bonds by Syn::P_*cpc*_YqjM belongs to the fastest reactions reported in cyanobacteria to date. This makes them excellent model reactions to investigate at which extent photosynthetic electrons can be deviated to heterologous production processes, and to identify and overcome limitations on the way to maximal productivity. While YqjM is undoubtedly one of the best characterised and most active representatives of ene-reductases, we aimed for the demonstration that *Synechocystis* is a proficient platform for different ene-reductases. Belonging to class III, YqjM differs from other members of the family in several ways such as the substrate scope^2^ and the oligomeric state.^26^ Therefore, we chose four more ERs from different classes and successfully produced them in recombinant *Synechocystis*. We tested our strains with a set of substrates (Fig. 2A) for their activity and selectivity. To our delight, all strains were active and specific activities of some strains were higher compared to previously characterised Syn::P_*cpc*_YqjM (Table 1 and Fig. 2B, C). With a few exceptions, the substrate scope met the reported substrate spectra of the isolated enzymes, showing that transport limitations are not crucial for the investigated substrates.^2,28,31,39^

**Table 1:** Light-driven whole-cell reduction of 1a–6a

Substrate	Strain	Conversion^a^ [%]		Yield^a^ [%]		Ketoreduction^b^ [%]		ee [%]		*A*_spec_ [U g_CDW_^−1^]	*A*_spec_ [U mg_chl_^−1^]
		1 h	24 h	1 h	24 h	1 h	24 h	1 h	24 h		
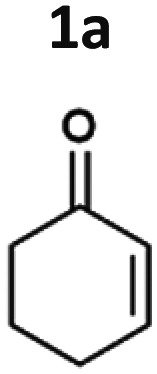	Syn::P_*cpc*_GluER	45.7 ± 3.7	>99.9	38.7 ± 4.3	>99.9	0.3 ± 0.05	42.1 ± 2.9	—	—	22.3 ± 0.6	1.2 ± 0.02
	Syn::P_*cpc*_OYE2	37.8 ± 3.5	>99.9	31.3 ± 4.5	>99.9	0.3 ± 0.03	34.4 ± 1.9	—	—	16.6 ± 1.9	0.9 ± 0.1
	Syn::P_*cpc*_OYE3	91.1 ± 0.2	98.5 ± 2.2	90.7 ± 4.9	>99.9	n.d.	32.4 ± 2.4	—	—	57.8 ± 0.8	3.5 ± 0.1
	Syn::P_*cpc*_YqjM	89.6 ± 2.7	>99.9	86.9 ± 7.0	88.7 ± 10.5	n.d.	19.5 ± 9.5	—	—	56.2 ± 5.0	3.1 ± 0.2
	Syn::P_*cpc*_TsOYE_2M	8.7 ± 3.2	82.8 ± 3.4	8.2 ± 0.6	72.4 ± 14.1	n.d.	11.3 ± 1.0	—	—	5.2 ± 0.8	0.3 ± 0.0

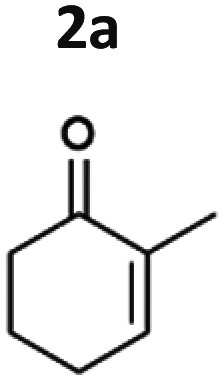	Syn::P_*cpc*_GluER	35.8 ± 1.8	>99.9	25.6 ± 2.0	76.2 ± 3.1	0.6 ± 0.1	55.0 ± 3.6	89 (*R*)	81 (*R*)	25.4 ± 2.5	1.3 ± 0.1
	Syn::P_*cpc*_OYE2	40.1 ± 1.2	>99.9	19.8 ± 3.2	63.0 ± 0.8	0.7 ± 0.1	46.5 ± 1.8	92 (*R*)	83 (*R*)	17.7 ± 3.4	0.9 ± 0.1
	Syn::P_*cpc*_OYE3	29.3 ± 3.4	>99.9	17.9 ± 1.6	69.9 ± 1.8	0.1 ± 0.1	59.4 ± 2.9	93 (*R*)	61 (*R*)	14.9 ± 1.1	0.9 ± 0.1
	Syn::P_*cpc*_YqjM	35.8 ± 8.4	>99.9	21.9 ± 1.3	95.2 ± 3.8	1.0 ± 0.5	61.4 ± 0.35	94 (*R*)	84 (*R*)	22.7 ± 2.9	1.1 ± 0.03
	Syn::P_*cpc*_TsOYE_2M	38.0 ± 01.5	99.9 ± 0.1	26.8 ± 0.7	67.0 ± 1.1	n.d.	27.2 ± 1.1	13 (*S*)	50 (*S*)	16.6 ± 0.9	0.9 ± 0.03

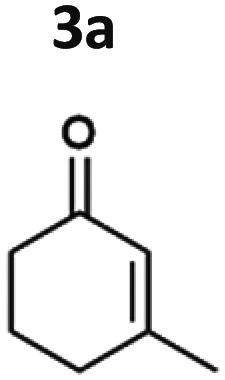	Syn::P_*cpc*_GluER	n.d.	n.d.	n.d.	n.d.	n.d.	n.d.	n.d.	n.d.	n.a.	n.a.
	Syn::P_*cpc*_OYE2	n.d.	4.4 ± 1.1	n.d.	5.1 ± 1.1	n.d.	n.d.	n.d.	>99 (*S*)	n.a.	n.a.
	Syn::P_*cpc*_OYE3	n.d.	n.d.	n.d.	4.8 ± 0.9	n.d.	n.d.	n.d.	>99 (*S*)	n.a.	n.a.
	Syn::P_*cpc*_YqjM	n.d.	n.d.	n.d.	n.d.	n.d.	n.d.	n.d.	n.d.	n.d.	n.d.
	Syn::P_*cpc*_TsOYE_2M	n.d.	26.9 ± 1.7	n.d.	13.4 ± 1.6	n.d.	n.d.	n.d.	65 (*S*)	n.a.	n.a.

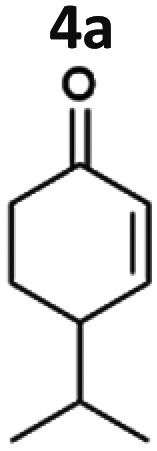	Syn::P_*cpc*_GluER	44.3 ± 3.5	56.3 ± 4.6	29.3 ± 4.4	38.0 ± 3.2	2.0 ± 0.6	7.9 ± 0.7	—	—	24.9 ± 0.9	1.3 ± 0.05
	Syn::P_*cpc*_OYE2	70.6 ± 1.7	99.9 ± 0.1	48.3 ± 4.1	66.6 ± 8.4	2.3 ± 0.3	6.9 ± 4.1	—	—	31.5 ± 2.4	1.9 ± 0.1
	Syn::P_*cpc*_OYE3	>99.9	98.7 ± 0.9	63.5 ± 2.3	68.2 ± 0.6	1.3 ± 0.3	2.3 ± 0.3	—	—	107.7 ± 1.6	5.9 ± 0.1
	Syn::P_*cpc*_YqjM	32.8 ± 0.6	68.0 ± 4.4	24.4 ± 0.2	47.8 ± 2.7	1.2 ± 0.4	5.6 ± 0.4	—	—	16.6 ± 0.8	0.9 ± 0.04
	Syn::P_*cpc*_TsOYE_2M	7.7 ± 3.3	17.2 ± 5.4	1.8 ± 0.2	2.2 ± 0.4	n.d.	0.7 ± 0.1	—	—	n.a.	n.a.

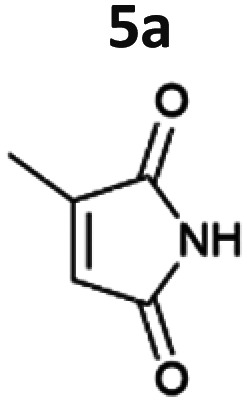	Syn::P_*cpc*_GluER	57.2 ± 1.7	>99.9	46.4 ± 2.5	72.1 ± 2.3	—	—	>99 (*R*)	>99 (*R*)	35.7 ± 2.6	1.6 ± 0.6
	Syn::P_*cpc*_OYE2	46.1 ± 2.4	>99.9	42.9 ± 4.0	98.1 ± 3.1	—	—	81 (*R*)	81 (*R*)	27.4 ± 3.8	1.3 ± 0.2
	Syn::P_*cpc*_OYE3	99.9 ± 0.1	>99.9	>99.9	53.9 ± 3.0	—	—	85 (*R*)	82 (*R*)	154.7 ± 3.3	8.2 ± 0.9
	Syn::P_*cpc*_YqjM	>99.9	>99.9	95.0 ± 7.2	86.7 ± 11.9	—	—	>99 (*R*)	>99 (*R*)	79.6 ± 4.1	3.16 ± 0.17
	Syn::P_*cpc*_TsOYE_2M	>99.9	>99.9	>99.9	>99.9	—	—	>99 (*R*)	>99 (*R*)	91.9 ± 28.3	4.4 ± 1.1

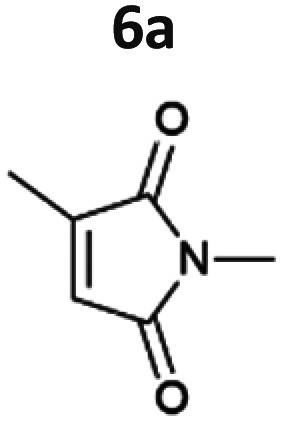	Syn::P_*cpc*_GluER	32.6 ± 1.4	>99.9	28.1 ± 1.6	>99.9	—	—	>99 (*R*)	99 (*R*)	14.8 ± 0.5	0.7 ± 0.1
	Syn::P_*cpc*_OYE2	63.6 ± 3.8	>99.9	63.1 ± 6.2	>99.9	—	—	>99 (*R*)	>99 (*R*)	35.6 ± 5.1	1.7 ± 0.4
	Syn::P_*cpc*_OYE3	>99.9	>99.9	>99.9	>99.9	—	—	>99 (*R*)	94 (*R*)	103.4 ± 19.2	6.3 ± 1.4
	Syn::P_*cpc*_YqjM	>99.9	>99.9	>99.9	>99.9	—	—	>99 (*R*)	>99 (*R*)	98.9 ± 3.1	5.3 ± 0.8
	Syn::P_*cpc*_TsOYE_2M	>99.9	>99.9	>99.9	>99.9	—	—	>99 (*R*)	92 (*R*)	138 ± 15.8	10.7 ± 1.4

a based on GC-FID chromatograms, yields include all possible products;

b observed reduction of products by the native enzyme pool in *Synechocystis*, n.a. = not analysed, n.d. not detected., *rac*-7a was excluded from the table since its high toxicity hindered reliable quantification of photobiotransformations.

In the reduction of cyclohexenone 1a, the highest activities were observed in Syn::P_*cpc*_YqjM and Syn::P_*cpc*_OYE3, while the activity of the other strains was low. In their review, Scholtissek *et al.*^2^ compared the conversion of several substrates by purified ERs from different classes. Regarding substituted cyclohexenone derivatives, authors concluded that α-substituted 2-methylcyclohexenone 2a is better accepted by ERs from class I and II which was not the case in *Synechocystis*. Unexpectedly, Syn::P_*cpc*_OYE3 and Syn::P_*cpc*_YqjM belonging to class II and III, respectively, were the enzymes with the highest whole-cell activity. After 1 h of reaction, the enantiomeric excess values (ee) for 2-methylcyclohexanone 2b correspond to those reported in the literature with 92% (*R*)^28^ and 93% (*R*).^39^ However, the enantiopurity of the product considerably decreased over time in all reactions. A similar racemisation of α-methyl ketone had been previously observed for levodione in *Synechocystis*.^22^

The intrinsic acidity of the α-C–H bond appears to lead to racemisation under the reaction conditions in the illuminated cell. Furthermore, reduction of 2b to the corresponding alcohols by intracellular *Synechocystis* alcohol dehydrogenases (ADHs) was observed. This ketoreduction becomes more pronounced with the reaction time and reached up to 59% (Syn::P_*cpc*_OYE3) after 24 h. As the enantiomer (*R*)-2b is predominantly converted by the intracellular enzymes, reduction to the alcohol also contributed to the observed decrease of the ee. In contrast to other strains, Syn::P_*cpc*_coTsOYE_2M was (*S*)-selective for the conversion of 2a. Here we observed an *enantio*-enrichment, albeit leading only to moderate optical purity. The detected 14% (*S*) ee after 1 h increased to 50% (*S*) after 24 h due to the preferred reduction of (*R*)-2b to the corresponding alcohol. Surprisingly, conversions for 3-methylcyclohexenone 3a were very low (not detectable after 1 h) with best conversions achieved for Syn::P_*cpc*_TsOYE_2M (13% yield after 24 h). Although it is known that β-substituted cyclic enones are less well accepted by ERs and not accepted by YqjM and TsOYE,^2^ good conversions were reported for OYE2 (up to *C* = 91%),^28^ OYE3 (up to *C* = 43%)^28^ and the TsOYE variant (up to *C* = 49%)^31^*in vitro*. No visible signs of toxicity were observed during biotransformation and reasons for this unexpected result remain unknown. As little is known about the facilitated transport of hydrophobic molecules into the autotrophic cyanobacterium, transport limitations cannot be excluded.

The substrate 4-isopropylcyclohexenone 4a is of particular interest as it is a potential intermediate for the synthesis of biobased polyesters. In 2017, Quilter *et al.* postulated a synthesis route for biodegradable polymers *via*4a from abundant β-pinene as starting material (Fig. 2D).^40^ Chemical synthesis steps included the ozonolysis and isomerisation of β-pinene followed by the rhodium-mediated hydrogenation of 4a to 4-isopropylcyclohexanone 4b. Subsequently, Baeyer–Villiger oxidation^41^ of 4b yields the corresponding lactone to produce 4-isopropyl-ε-caprolactone as monomer of biodegradable polyesters.^40^ We envisioned to replace the rhodium-mediated hydrogenation by more sustainable light-driven bioreduction. To our knowledge 4a was never investigated as a substrate for ERs before and we were pleased to see that it was well accepted by our strains. Syn::P_*cpc*_OYE3 completely reduced 10 mM 4a within 1 h and the high activity of 107 U g_CDW_^−1^ underlines the efficiency of the reaction. In fact, this is the first time we have observed such high cyanobacterial whole-cell activity for a substrate other than maleimides. Furthermore, reduction of 4b to the corresponding alcohols *cis*- and *trans*-4-isopropylcyclohexanol 4c was negligible.

Our results confirm the high activity and selectivity of ERs from all classes on maleimides like 2-methylmaleimide 5a and 2-methyl-*N*-methylmaleimide 6a. Although initial rates with Syn::P_*cpc*_OYE2 and Syn::P_*cpc*_GluER were considerably lower compared to the other strains, both fully converted 5a and 6a within 24 h.

The strains Syn::P_*cpc*_OYE3 and Syn::P_*cpc*_coTsOYE_2M were even more active on 5a and 6a compared to Syn::P_*cpc*_YqjM. While the class I and class III enzymes showed excellent selectivity for 5a, ee values were moderate for the investigated class II enzymes.

Beside standard substrates for asymmetric alkene reductions, we were interested in integrating substrates with potential industrial relevance to our screening. Citral 7a is the first acyclic ketone investigated in our set-up and one of the products, (*R*)-citronellal (*R*)-7b, is a precursor to produce the fragrance (–)-menthol.^42^ In particular, OYE2 exhibits the desired selectivity and was recently applied in a cascade reaction to produce (*R*)-7b.^29^ Unfortunately, light-driven reduction of 7a was unsuccessful and could not be quantified reliably. Obtained yields for all strains were low with Syn::P_*cpc*_OYE3 reaching best concentrations of 0.6 mM 7b after 1 h. Discolouration of the cells from green to blue after 24 h of incubation indicated toxicity of 7a as the apparent reason (Fig. S5†). Furthermore, loss of substrate and products due to their volatility and the appearance of side-products rendered this substrate unsuitable for light-driven biotransformations in *Synechocystis*.

For the other substrates, the remarkably high activity of Syn::P_*cpc*_OYE3 can be explained by the high concentration of OYE3 inside the cell. Western Blot analysis (Fig. S2†) clearly show highest protein levels of the soluble ERs compared to the other strains under the same cultivation conditions. In contrast, the concentration of GluER seemed significantly lower as the band only appeared faintly. This could explain the moderate activity of Syn::P_*cpc*_GluER.

It should be noted that the mass balances after 24 h do not match the initial substrate concentration for reactions with 2a, 4a, 5a and 7a (Fig. 2E and Fig. S6–S10†). This was either attributed to evaporation of compounds during sampling (in case of 2a and 7a) or to an unknown depletion of the product (in case of 5b). The latter can be circumvented by stopping the reaction when full conversion is reached. Concentration of 4b remains stable over time and both evaporation and metabolisation by the cell were excluded (Fig. S12†). The reason for the 32% loss of compounds in the mass balance remains elusive.

### Up-scale of the photobiotransformation in a flat panel photobioreactor

Adequate light availability is a main challenge for the up-scaling of light-driven biotransformations. The absorption of light by the cells creates a gradual decay of illumination in the reaction vessel in the direction opposite to the light source, also known as the self-shading effect.^43^ As cell densities and the diameter of the reaction vessel increase, light penetration decreases, causing only cells near the irradiation source to receive adequate light.^42^ Under high cell density, especially in the reaction volumes with a longer optical path, self-shading leads to the formation of dark regions within the reactor and therefore reduces the efficiency of the reaction.^44^ Thus far, reactions catalysed by Syn::P_*cpc*_YqjM have been investigated in three different reactor set ups. The bubble column reactor (BCR) uses wireless light emitters for internal illumination.^33^ The highest specific activity achieved for the BCR was reported with 65.5 U g_CDW_^−1^ at 1.2 g_CDW_ L^−1^. However, with an increase in cell density, the self-shading effects becomes prevalent, and the specific activity decreases to approximately 33 U g_CDW_^−1^ at 2.4 g_CDW_ L^−1^ (Table 2). In comparison, the coil reactor seems to be the best system regarding efficiency, since the 2 mm inner diameter of the pipes allows optimal external illumination. Indeed, the cell density within the coil reactor could be increased to 4.8 g_CDW_ L^−1^ without substantial loss of specific activity.^34^ However, with an active illuminated volume of <5 mL, the scale-up of the coil reactor needs to be performed by parallelizing reactor set-ups (numbering-up) to prevent oxygen accumulation and pH gradients.^45,46^ Another reactor concept in the form of the illuminated stirred tank reactor (STR) is unfavourable for photobiotransformations since the high diameter of the reaction vessel limits the light availability inside the tank. Consequently, the applied cell density on a larger scale is restricted which results in low volumetric productivities.^33^ Nevertheless, the STR was successfully used for several oxyfunctionalisations^16–18,32^ with product yields up to the gram-scale. Limitations such as substrate toxicity or inhibition were alleviated by utilizing a two-phase system^16^ to extract the product or *via* substrate feeding.^5,33,34^

**Table 2:** Upscaling of the light-driven reduction of 10 mM 5a. Data obtained from the novel flat panel reactor are compared to data in literature for the bubble column reactor (BCR), the continuous stirred tank reactor (CSTR) and the coil reactor

Reactor	Strain	*T* [°C]	Volume [mL]	Cell density [g_CDW_ L^−1^]	*P* [%] 1 h	Initial rate^b^ [mM h^−1^]	*A*_spec_ [U g_CDW_^−1^]	STY [g L^−1^ h^−1^]	Ref.
FPBR	Syn::P_*cpc*_OYE3	RT	120	2.4	89.3	8.1	56.1	0.9	This study
				3.6	>99.9	9.0	41.9	0.9	
	Syn::P_*cpc*_YqjM			2.4	73.9	6.6	46.2	0.7	
				3.6	95.4	8.4	38.8	1.0	
	Syn::P_*cpc*_TsOYE_2M			2.4	87.0	7.4	51.1	0.9	
				3.6	89.4	7.3	33.6	0.9	

BCR	Syn::P_*cpc*_YqjM	30	200	2.4	∼42^b^	3.7	32.5	0.3	33

CSTR	Syn::P_*cpc*_YqjM	RT	4.7^a^	3.6	∼80^b^	6.0	28.5	4.1	34

Coil	Syn::P_*cpc*_YqjM	30	4.7^a^	3.6	>99.9	21.6	99.8	14.4	34
		25		3.6	>99.9	13	60.7	8.5	
		25		4.8	>99.9	16^b^	58.7	n.d.	

a illuminated, active volume. Total volume of the reactions was 15 mL and not illuminated volume stayed in a reservoir [*b*] values were estimated based on graphs in the respective publications.

b based on product formation, T = temperature, P = product formation, A_spec._ = specific activity, STY = space time yield, n.c. = not calculated, RT = room temperature.

**Fig. 3:**
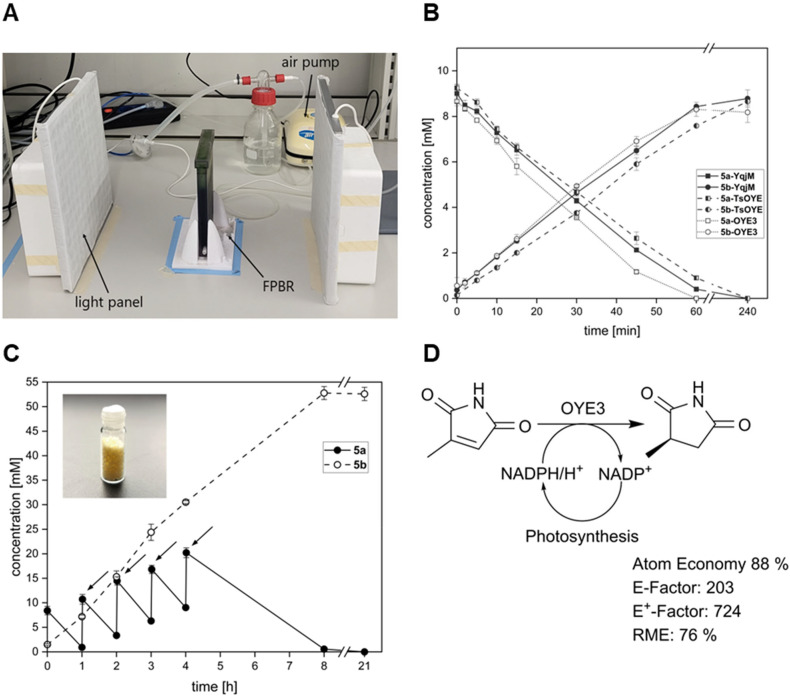
Photobiotransformations in the flat panel photobioreactor (FPBR). (A) Set-up of the reactor; (B) conversion of 10 mM 5a and formation of 5b in the FPBR by three different *Synechocystis* strains harbouring recombinant ene-reductases YqjM, OYE3 or TsOYE_2M; (C) conversion of 5a and formation of 5b in the FPBR for the fed-batch reaction (50 mM) with an obtained yield 591.0 mg (inset) with three technical replicates and (D) light-driven ene-reduction of 5a in recombinant cyanobacteria with associated sustainability metrics. Reaction conditions: cell concentration = 3.6 g_CDW_ L^–1^, 300 μmol_photons_ m^−2^ s^−1^ (Roleandro, HY-MD-D169-S-75W-RB LED panel), *V* = 120 mL, results include data of three technical replicates.

Recently, Tüllinghoff *et al.*^32^ produced 23.5 mM 6-hydroxyhexanoic acid within 48 h in a two-step cascade. While 3.7 g_6−HA_ g_CDW_^−1^ remains an impressive result for an oxyfunctionalisation reaction, the low volumetric productivity of the reaction indicated a strong self-shading effect in the employed stirred-tank reactor. Hence, we designed a flat panel photobioreactor (FPBR) to achieve efficient light distribution with air mixing. The designed template was 3D printed as a whole unit using a transparent biocompatible photocurable resin (Fig. 3A). The FPBR offers a compromise between a reduced optical path (1 cm) as seen in the coil reactor and the reactor volume of an STR or a cylindrical BCR. Due to its low thickness and high-surface-to-volume ratio of 310 m^2^ m^−3^, it enables more effective lighting even with external illumination.

In small-scale photobiotransformation reactions, both Syn::P_*cpc*_OYE3 and Syn::P_*cpc*_TSOYE_2M have shown higher activity for the conversion of maleimides 5a and 6a than the well-characterised Syn::P_*cpc*_YqjM. Therefore, we decided to investigate the reduction of 5a by all three strains in the FPBR with two different operating cell densities (2.4 g_CDW_ L^−1^ and 3.6 g_CDW_ L^−1^). With up to 56.1 U g_CDW_^−1^, Syn::P_*cpc*_OYE3 was most active but closely followed by Syn::P_*cpc*_TSOYE_2M and Syn::P_*cpc*_YqjM (Table 2). This confirms the trend observed for small scale reactions. High yields up to >99% after 1 h for reactions with 3.6 g_CDW_ L^−1^ (Fig. 3B) and initial rates of 6.6 to 9.0 mM h^−1^ further proved the efficiency of biotransformation in the FPBR. Furthermore, STY close to 1 g L^−1^ h^−1^ were achieved for multiple strains and cell densities. This surpasses values reported for the BCR by threefold and, to the best of our knowledge, represents the highest STY for light-driven biotransformation to date for volumes exceeding 100 mL. To further support the feasibility of our reactor system, we chose to increase concentration of 5a to 50 mM. To mitigate the toxic effects of 5a,^5^ we adopted a fed-batch strategy, wherein 10 mM of 5a were added every hour until reaching a total of 50 mM (Fig. 3C). We opted for the setup that demonstrated the highest performance, as determined by the rate of product formation (Syn::P_*cpc*_OYE3, 3.6 g_CDW_ L^−1^).

After 8 h, 52.8 mM 5b was produced with 0.6 mM of 5a left as determined by GC-analysis. At the 21 hour mark, the reaction was terminated and subjected to product isolation. The successful conversion of 50 mM resulted in 591.0 mg 5b formation and a specific isolated yield of 87% after organic phase extraction. With our reactor system, we report product concentration of 4.9 g L^−1^ within 8 hours. The purity of the product was confirmed by ^1^H NMR (Fig. S14†). Scaling-up practices for pilot or industry scale FPBRs include increasing the height and width of the FPBR.^37^ Extending the depth and thus the light path need to be carefully weighed with deployed cell concentration. While we could show that molar productivities increase accordingly with rising cell density, the efficiency, denoted by specific activity, tends to decline as cell densities increase. Thus, self-shading effects may still be a limiting factor for exploiting the full potential of the FPBR.

### Sustainability metrics of photobiotransformations

The sustainability of whole-cell photobiotransformations is frequently highlighted, as photoautotrophic organisms require minimal resources for growth, fix carbon dioxide, and produce oxygen. Furthermore, photosynthesis can be used to efficiently recycle cofactors for redox reactions as presented in this study and others.^15,22,32^ By utilizing water as the electron donor, the need for sacrificial cosubstrates is eliminated which improves the atom economy and facilitates down streaming processes. However, examples that quantify these advantages using comparable metrics are rare. Therefore, we decided to evaluate the sustainability of our reaction set-up to set a benchmark value for future biocatalytic processes. In particular, we here consider the conversion of 5a in the FPBR which yielded 591 mg (87%) isolated product with high purity. In the following, we discuss three key metrics to categorise the sustainability of our process.

The atom economy addresses the overall efficiency of a chemical reaction by evaluating the proportion of reactants that are incorporated into the desired product.^47^ An advantage of whole-cell biotransformations is the capability of the host organism to regenerate cofactors *in situ* eliminating the need for expensive and stoichiometric addition of cofactors.^48^ In contrast, cofactor recycling in heterotrophic hosts is usually driven by the use of sacrificial co-substrates like glucose or formate.^49^ This potentially reduces the atom economy of the process, as only part of the electrons stored in the molecule are used for NADPH recycling.^6^ Accordingly, we calculated atom economies reaching 49% for ene-reductions in heterotrophic hosts using glucose as co-substrate.^50,51^ The precise measure to which electrons from glucose are directed towards the biotransformation in whole-cell bioprocesses remains unknown. In growing cells, it is assumed that approximately 12% of glucose is channelled through the oxidative pentose phosphate pathway in *E. coli* regenerating two NADPH per glucose molecule.^52^ Furthermore, other enzymes from the tricarboxylic acid cycle, glycolysis and outside the central carbon metabolism contribute to NADPH recycling. Additionally, most ene-reductases promiscuously also accept NADH that stems from various glycolytic pathways.^3–5^ In practice, an excess amount of glucose is typically supplied to compensate for metabolic losses.

**Fig. 4:**
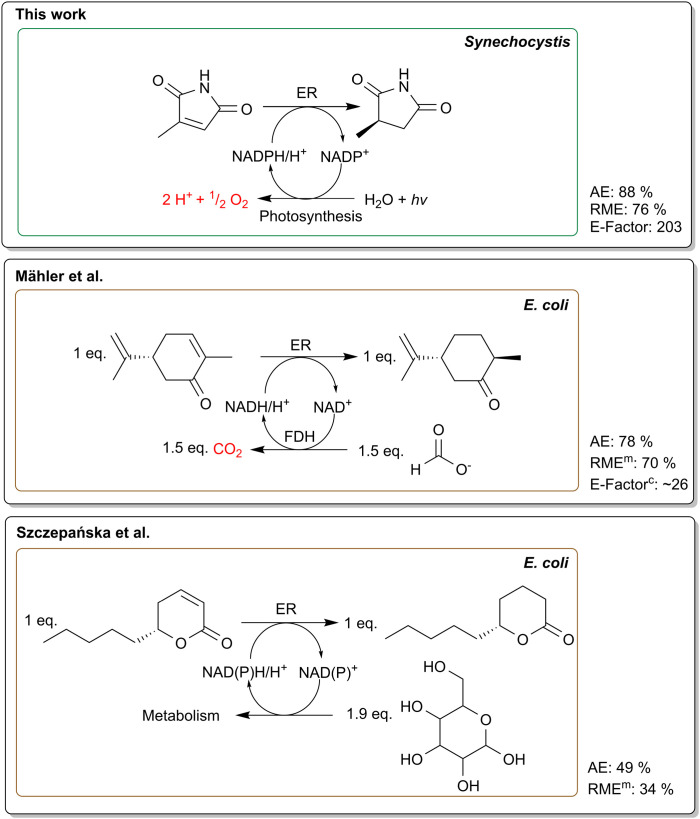
Comparison of common NADPH recycling methods using recombinant *E. coli* versus photosynthesis-based NADPH regeneration. Formate dehydrogenase (FDH) based recycling system is represented by Mähler *et al*.^55^ whereas Szczepańska *et al*.^51^ shows a reaction with a glucose driven regeneration system. Furthermore, the reaction mass efficiency (RME), atom economy (AE) and *E*-factor are shown. For the studies where no product was isolated, the measured product concentration was used (RME^m^). In addition, we calculated the *E*-factor for the Mähler *et al*.^55^ reaction (*E*-factor^c^) according to the information from their paper. For Szczepańska *et al*.^51^ no *E*-factor was calculated due to the possible contortion of using a low volume (1 mL). Photosynthetic NADPH regeneration offers the highest atom economy, eliminates the need for sacrificial co-substrates and produces O_2_.

Another route to regenerate cofactors is to use a two-enzyme system for whole-cell ene-reductions, *e.g.*, employing formate dehydrogenase.^53–56^ The low molecular weight of formate generally improves the atom economy reaching up to 78%.^55,56^ While this shows a substantial improvement of atom economies, use of water as an electron donor is even more favourable (Fig. 4). Water splitting to generate electrons is enabled by employing photosynthetic *Synechocystis* as hosts for biotransformations.^6,19^ Since water is abundant in the medium, it eliminates the need for sacrificial substrates completely. As described previously, the reaction of 2-methylmaleimide to 2-methylsuccinimide offers an excellent atom economy of 88%^22^ and the atom economies for the very similar substrates 1a–6a range from 86%–90%. Interestingly, the light-driven enzymatic Baeyer–Villiger oxidation of cyclohexanone as reported previously^13,18,57^ reaches an AE of 98%, under the assumption that photosynthetically produced oxygen is incorporated into the product.

Compared to atom economies, the Reaction Mass Efficiency (RME) provides a more comprehensive assessment by accounting for both the stoichiometric balance and the actual yield of the reaction.^58^ Constable *et al.*^59^ calculated RMEs for 28 different chemistries ranging from 83% for acid salt chemistry to 27% for *N*-dealkylations. With an RME of 76%, our process is among the most efficient reactions and has a slightly higher RME compared to chemical hydrogenations (RME = 74%).^59^ However, it is important to note that these hydrogenation reactions typically require the use of heavy metals, which pose significant environmental concerns, whereas enzymatic transformations offer potent tools for producing compounds with excellent stereoselectivity.^60,61^ We were unable to find RME values for other whole-cell reductions in literature. Since many studies do not focus on product isolation, we calculated the RMEs of comparable studies^51,55,56^ using the measured product (RME^m^) (Fig. 4). In a noteworthy study, Rapp *et al.*^56^ optimised the bioreduction of *o*-chloroacetophenone by using recombinant *E. coli* and cyclodextrins to enhance solubility of the substrate. We calculated an RME^m^ of 77% for the reaction, slightly outperforming our reaction. However, due to unoptimised extraction, the actual RME of the same study dropped to 65% after product isolation. In contrast, a substantially lower RME^m^ of 34% was calculated for the glucose-driven whole-cell reduction of massoia lactone described by Szczepańska *et al.* (Fig. 4).^51^

The *E*-factor, in contrast, quantifies the efficiency of a process by comparing the mass of waste generated to the actual mass of the product obtained.^62^ Thus, a process with zero waste produced will have an *E*-factor of 0. Since *E*-factors are additive, they can be calculated for each individual step, facilitating the identification of primary waste contributors.^62^ In industrial processes, an *E*-factor up to 5 is considered acceptable for bulk chemicals, up to 20 for fine chemicals, and up to 100 for high-value products and pharmaceuticals.^62^

We were able to obtain an *E*-factor of 203 for our fed-batch experiment which is, to the best of our knowledge, the first complete *E*-factor reported for cyanobacterial photobiotransformations (Table 3). Since the volumetric productivities of heterologous ene-reductions in *Synechocystis* are among the highest observed in cyanobacterial production, we believe, that our *E*-factor sets a benchmark value for evaluating the sustainability of cyanobacteria as production platforms in future. Furthermore, it shows clear directions on how to improve the sustainability of such systems.

**Table 3:** *E*-factors for the 50 mM fed-batch experiment

	sEF^a^	*E*-Factor	*E* ^+^-Factor^b^	CO_2_^c^ (%)
Cultivation	4.3	1521	9342	84
Biotransformation	1.3	203	724	72
Total	5.6	1724	10 066	83

a Exclusion of water.

b Inclusion of electricity usage as 242 g_CO_2__ kW h^−1^.^63^

c Percentage of CO_2_ measured from the total *E*^+^-factor.

Strategies to improve the *E*-factor include reducing the applied water amount which accounts for a major part of waste formed. It should be noted, however, that the potential impact on global warming associated to waste disposal can differ considerably. In the best case, wastewater can be treated under mild conditions in a treatment plant. However, in many chemical processes, pre-treatment or even burning of wastewater is necessary to prevent the release of hazardous chemicals into the environment.^64^ In any case, the impact of wastewater on the *E*-factor should not be neglected.^7^ This is underlined by the simple *E*-factor (sEF) which excludes water and solvents from the calculation. The comparison of the sEF of our reaction (1.3) with the complete *E*-factor (203) shows the potential room for improvement (Table 3). In future, the re-use of immobilised cells^35,65,66^ could reduce the amount of water required for the cultivation considerably.

As indicated by the comparison between the *E*-factor and the sEF, media components and cell weight contribute only minimally to the overall composition of the *E*-factor (Table 3).

From the 3.3 g waste produced (excluding water), 0.43 g was contributed by cells that grew autotrophically from using water and atmospheric CO_2_. Therefore, only marginal improvements can be attained by reducing the cell quantity, as the proportion of non-water waste remains below 0.3%. Thus, the *E*-factor of the coil reactor^34^ or the bubble column reactor^33^ differs negligibly from the FPBR if it is adjusted for employed substrate concentration with *E*-factors of 232 and 231, respectively. More impactful improvements can be realised by increasing the relatively low substrate concentration of 50 mM. For an *E*-factor of 20, the substrate concentration would need to be increased about 10-fold (500 mM), and for 5 about 40-fold (2 M). Since toxic effects of high substrate concentrations are often observed for whole-cell biotransformations and particularly for cyanobacteria^5,12,67^ and in the current study (Fig. S5†), reactions would need to be performed as a fed-batch approach or by using immobilised cells in a continuous system.

The positive impact of higher volumetric yields can be clearly seen in a recent study of Mähler *et al.*^55^ where they used a formate dehydrogenase system to regenerate the cofactor for the reduction of 300 mM (*R*)-carvone in 0.7 L scale using whole-cell *E. coli* harbouring an ene-reductase from *Nostoc* sp. Albeit having a lower atom economy of 70%, they obtained an *E*-factor of 26 which is 10 times lower than our cyanobacterial process.

Nevertheless, we note that the rate of 616 mg h^−1^ L^−1^ obtained in our process compares very favourably to the 65–226 mg h^−1^ L^−1^ that were obtained in photobiotransformations in other photobioreactors,^18,32–34,68^ and to the 0.06–16 mg h^−1^ L^−1^ that were obtained in photoproduction processes in cyanobacteria.^69–77^

Finally, we propose that power consumption should be considered an important metric for assessing the environmental sustainability of processes in light-driven biotransformations. In 2019, Tieves *et al.* proposed an addition to the traditional *E*-factor in the form of the *E*^+^-factor which includes the electricity usage by converting power into CO_2_ equivalents.^78^ Currently in Europe, the production of one kW h electricity generates an average of 242 g CO_2_.^63^ Thus, *E*^+^-factor values are significantly higher than *E*-factors, primarily due to the substantial mass of CO_2_ generated during electricity production. We report an *E*^+^-factor of 724 for the biotransformation and an *E*^+^-factor of 10 066 when cultivation is included. We were unable to find further *E*^+^-factor values for biocatalytic redox transformations in the literature except for a very insightful study published by Tieves *et al.*,^78^ where an *E*^+^-factor of approximately 100 000 for the lab-scale production of unspecific peroxygenases (UPO) was reported. Similar *E*^+^-factor values were also calculated to produce a formate oxidase in 10 l-scale using *Pichia pastoris*.^78^

For cyanobacterial production processes, constant illumination during biotransformations is a major source of energy consumption. Indeed, in our fed-batch process, 8 hours of illumination at 300 μmol_photons_ m^−2^ s^−1^ accounted for 94% of the total electricity consumption during the biotransformation (Table S5†). Tüllinghoff *et al.* (2023) recently reported the gram-scale synthesis of lactones using an illumination of 700 μmol_photons_ m^−2^ s^−1^ in the span of 2 days for photobiotransformation.^32^ Similarly, Miao *et al.* reported photoproduction of 435 mg L^−1^ isobutanol over 40 days with a light intensity of 50 μmol_photons_ m^−2^ s^−1^.^69^ These examples illustrate that cyanobacterial production processes often require high light intensities or extended periods of irradiation. Therefore, we conclude that the length and the intensity of electrical illumination are substantial factors for the sustainability of any cyanobacterial production process.

However, the biotransformation itself is only responsible for 7% of the *E*^+^-factor (Table 3). The main contributor thereof is the cultivation of *Synechocystis*. The growth of *Synechocystis* tends to be slow and is highly dependent on the cultivation setup (here *μ* = 0.70 ± 0.13 d^−1^). Our current setup in aerated glass tubes is clearly not optimised for minimal electricity consumption; therefore, we see significant potential for the up-scaling of the cultivation conditions towards more efficient flat panel systems which is expected to reduce electricity usage by lowering the light intensity or omitting the use of the thermostat. This needs to be carefully balanced with presumably elongated cultivation time. On a larger scale, sunlight may in principle substitute the need for illumination during the day. However, this renders the process susceptible to weather fluctuations and seasonal changes which in turn hinders the overall growth rate. Recently, several fast-growing and salt-tolerant cyanobacterial strains have been isolated^79^ and present themselves as a possible solution for this problem.

## Conclusion

Light-driven biotransformations in cyanobacteria have the potential to greatly improve the atom economy of redox biocatalysis. Here, we demonstrate the intensification of a photobiotransformation by enzyme screening, enzyme production in cyanobacteria and up-scale in a flat-panel photobioreactor. OYE3 from class II turned out to be best produced in *Synechocystis* and the recombinant strain was most active reaching up to 155 U g_CDW_^−1^ in small scale. The substrate 2-methylmaleimide was successfully converted in a 120 mL batch with observed activities up to 56 U g_CDW_^−1^. The efficiency was highlighted by converting 50 mM of 2-methylmaleimide with an isolated yield of 87%_._ This marks a successful process with an atom economy of 88%. An analysis of *E*^+^-factor and atom economy led to the identification of crucial factors for the sustainability of this reaction. The substrate concentrations should be increased substantially to facilitate down-stream processing and reduce formation of aqueous waste. This will also require solutions to circumvent toxic effects of substrates and products, a common problem in whole-cell processes in general. Energy consumption for the illumination of the reaction calls for short reaction times – even in a cyanobacterial process as short as 8 h as CO_2_ formation associated to illumination was substantial. Finally, formation of aqueous waste and illumination during the cultivation are important factors. We believe that the sustainability analysis reported here will set a benchmark for assessing the sustainability of cyanobacterial photobiotransformations and highlights challenges that must be overcome in order to exploit the exceptionally high atom efficiency of the approach.

## Experimental

### Chemicals and reagents

All chemicals were purchased with the highest available purity from Carl-Roth GmbH (Karlsruhe, Germany), Merck (Darmstadt, Germany) or Thermo Fisher Scientific (Waltham, U.S.), unless otherwise stated. 2-Methylmaleimide was synthesised as described in previous studies.^22^

### Plasmid and strain construction

All integrative vectors were cloned using FastCloning.^80^ The respective templates and primers are listed in Tables S1 and Table S2.† The codon-optimised gene for variant TsOYE C25G I67 T (TsOYE_2 M) was ordered at Integrated DNA Technologies. All genes for ERs contain an *N*-terminal His-tag. They were set under control of the promoter P_*cpc*_ and integrated into the genome locus *slr0168* by homologous recombination. Verification of gene integrations were performed by gDNA isolation (High Pure PCR Template Preparation Kit, Roche) and subsequent segregation check *via* PCR. Furthermore, Western Blot analysis confirmed the presence of the recombinant ene-reductases (Fig. S2†). The used primers are listed in Table S2.† The integration of the gene for wildtype TsOYE into the genome of *Synechocystis* was not successful even after several attempts.

The plasmid pSHDY_P_*rha*_mVenus for rhamnose-inducible expression was designed as previously described^38^ and ordered from Addgene. The gene of the fluorescent protein was exchanged to *yqjm* using Gibson cloning. Transformation of *Synechocystis* sp. PCC 6803 with integrative and replicative plasmids was performed as described in previous studies.^5,12,38^

### Cultivation of cyanobacterial strains

*Synechocystis* sp. PCC 6803 wild type and recombinant strains harbouring genes for the ene-reductases were cultivated Erlenmeyer flasks (300 mL) in BG11 or on BG11 agar plates under constant white light (20–60 μmol_photons_ m^−2^ s^−1^) at 30 °C, 50% humidity and no additional CO_2_ supply. BG11 for most recombinant strains was supplemented with 50 μg mL^−1^ kanamycin. Only BG11 for strains harbouring the rhamnose-inducible promoter system was complemented with 40 μg mL^−1^ spectinomycin. Induction of this system was performed with rhamnose (10 mM) at OD_750_ = 0.5. Cells for reactions in the FPBR were cultivated in an aquarium as previously reported.^33^ For long time storage, cells were frozen in BG11 with 10% glycerol at −80 °C.

### Light-driven biotransformations

Light-driven biotransformations were performed as described previously.^5,12^ Cells were harvested at an optical density (OD) at 750 nm of 1.0–2.0 and concentrated to the final cell density (OD_750_ = 10), 2.4 g_CDW_ L^−1^ as determined previously.^5^ Under standard conditions, reactions (*V*_total_ = 1 mL, 30 °C, 130 rpm) were performed in glass vials (4 mL) under continuous light (150 μmol_photons_ m^−2^ s^−1^) in a self-built photobioreactor.^81^ The reactions were initiated by the addition of substrate (10 mM). Reactions with substrates 7a and 4a contained 2% (v/v) DMSO. Cell dry weight (CDW) and chlorophyll a (chl a) content were determined as described previously.^5^ Samples (100 μL) were taken after defined time points and the reactions were quenched with liquid nitrogen.

### Flat panel photobioreactor

The FPBR with 142 ml working volume and 18 mL headspace was 3D printed using the home-made template with 1 cm optical path, 16 cm height, and 10 cm width to form the working volume and headspace. The bottom part of the FPBR was designed for efficient two-side airlift mixing within the culture volume. The FPBR was fabricated of a transparent biocompatible photocurable polymer resin (BioMed Clear, Formlabs). A BOYU air-pump (S-4000B) was used for bubbling with an air flow rate of 0.5 L min^−1^. Red-blue light was provided by LED growth panels (Roleandro, HY-MD-D169-S-75W-RB) that were covered by parchment paper to reduce light intensity. The light intensity of 300 μmol_photons_ m^−2^ s^−1^in the reaction was measured by submerging the LI-COR photometer (LI-250A) probe in water filled FPBR and adjusted by altering the distance of the lights to the reactor. Reactions (120 mL) were performed in BG11 with *Synechocystis* cells concentrated to the desired OD_750_ and initiated by the addition of substrates (10 mM). The reaction suspension was mixed in the dark for 1 minute, before the lights were switched on and the first sample (*t* = 0 h) was taken.

### GC-FID analysis

Reactions with substrates 1a–4a and 7a were extracted with dichloromethane (300 μL) and reactions with substrates 5a and 6a were extracted with ethyl acetate (300 μL). *n*-Decanol or dodecane (2 mM) were used as internal standards (IST). Samples were centrifuged and the organic phase was dried with anhydrous MgSO_4_ before analysed *via* gas chromatography with flame ionisation detector (GC-FID). Unless otherwise specified, the sample (1 μL) was injected (*T* = 230 °C, split ratio = 20, purge flow = 3 mL min^−1^) and nitrogen was used as carrier gas. Detection was carried out at 320 °C with a sampling time of 40 ms and flow rates of 40 mL min^−1^ and 400 mL min^−1^ for H_2_ and synthetic air, respectively. Information about the used columns (Table S6†) and methods as well as reference chromatograms are listed in the ESI.†

### Organic phase extraction for preparative-scale FPBR

The cell suspension was centrifuged (3250*g*, 15 min) and the supernatant was carefully decanted from the cell pellet. A separating funnel was filled with 1.3 times the volume of ethyl acetate compared to the reaction suspension (160 mL). The separating funnel was vigorously shaken to allow for the partitioning of the organic compounds into the ethyl acetate phase. The extraction process was repeated three more times and subsequently, the organic phase was dried with anhydrous MgSO_4_. After filtering, the organic phase was transferred to a round bottom flask and vaporised by rotary evaporation under vacuum. To aid in the drying process, a few millilitres of dichloromethane were added to the flask. The evaporation process was repeated until the product was sufficiently dry.

### Electricity usage for *E*^+^-factor calculations

Electricity was measured by BEARWARE Power Meter (Model: 302717) taking the average electricity power usage after a 24 h measurement.

## Author contributions

H. C. G. coordinated, planned, and performed experiments, established the analytics, and wrote part of the manuscript. P. E. and H. M. performed experiments and were involved in their interpretation. Furthermore, P. E. performed all reactions in the flat panel photobioreactor and wrote part of the manuscript. L. M.-Y. performed and analysed biotransformations with Syn::P_*cpc*_TsOYE_2 M isolated and characterised the product after fed-batch experiments and improved the manuscript. L. S. cloned plasmids for the rhamnose promoter, performed the respective experiments and was involved into initial studies for the proposed cascade reaction. J. Z. cloned the integrative vectors for OYE2, OYE3 and GluER, performed first biotransformations and was involved in the method development for GC-FID analysis. C. E. P. helped to conceptualise the project and to interpret results. Furthermore, she provided the genes of OYE2, OYE3 and GluER. S. N. K. and Y. A. designed and fabricated the flat panel reactor and contributed to the review and editing of the manuscript. R. K. coordinated and supervised the project.

## Conflicts of interest

The authors declare that there are no conflicts of interest.

## Supplementary Material

GC-027-D4GC05686H-s001

## Data Availability

The data that supports the findings of this study can be found in the ESI.† Additional data (*e.g.*, raw data, spectra, *etc*.) are available upon request.

## References

[cit1] Toogood H. S., Scrutton N. S. (2018). ACS Catal..

[cit2] Scholtissek A., Tischler D., Westphal A. H., van Berkel W. J. H., Paul C. E. (2017). Catalysts.

[cit3] Robescu M. S., Niero M., Hall M., Cendron L., Bergantino E. (2020). Appl. Microbiol. Biotechnol..

[cit4] Knaus T., Paul C. E., Levy C. W., De Vries S., Mutti F. G., Hollmann F., Scrutton N. S. (2016). J. Am. Chem. Soc..

[cit5] Assil-Companioni L., Büchsenschütz H. C., Solymosi D., Dyczmons-Nowaczyk N. G., Bauer K. K. F., Wallner S., Macheroux P., Allahverdiyeva Y., Nowaczyk M. M., Kourist R. (2020). ACS Catal..

[cit6] Grimm H. C. and Kourist R., in Photosynthesis: Biotechnological Applications with Microalgae, ed. M. Rögner, De Gruyter, 2021, vol. 3, p. 57

[cit7] Ni Y., Holtmann D., Hollmann F. (2014). ChemCatChem.

[cit8] Medipally H., Mann M., Kötting C., van Berkel W. J. H., Nowaczyk M. M. (2023). ChemBioChem.

[cit9] Kramer D. M., Sacksteder C. A., Cruz J. A. (1999). Photosynth. Res..

[cit10] Malihan-Yap L., Grimm H. C., Kourist R. (2022). Chem. Ing. Tech..

[cit11] Jodlbauer J., Rohr T., Spadiut O., Mihovilovic M. D., Rudroff F. (2020). Trends Biotechnol..

[cit12] Büchsenschütz H., Vidimce-Risteski V., Eggbauer B., Schmidt S., Winkler C., Schrittwieser J. H., Kroutil W., Kourist R. (2019). ChemCatChem.

[cit13] Erdem E., Malihan-Yap L., Assil-Companioni L., Grimm H., Barone G. D., Serveau-Avesque C., Amouric A., Duquesne K., De Berardinis V., Allahverdiyeva Y., Alphand V., Kourist R. (2022). ACS Catal..

[cit14] Böhmer S., Marx C., Gómez-Baraibar Á., Nowaczyk M. M., Tischler D., Hemschemeier A., Happe T. (2020). Algal Res..

[cit15] Hoschek A., Bühler B., Schmid A. (2017). Angew. Chem., Int. Ed..

[cit16] Hoschek A., Bühler B., Schmid A. (2019). Biotechnol. Bioeng..

[cit17] Hoschek A., Toepel J., Hochkeppel A., Karande R., Bühler B., Schmid A. (2019). Biotechnol. J..

[cit18] Tüllinghoff A., Uhl M. B., Nintzel F. E. H., Schmid A., Bühler B., Toepel J. (2022). Front. Catal..

[cit19] Mascia F., Pereira S. B., Pacheco C. C., Oliveira P., Solarczek J., Schallmey A., Kourist R., Alphand V., Tamagnini P. (2022). Green Chem..

[cit20] Jurkaš V., Winkler C. K., Poschenrieder S., Oliveira P., Pacheco C. C., Ferreira E. A., Weissensteiner F., De Santis P., Kara S., Kourist R., Tamagnini P., Kroutil W. (2022). Eng. Microbiol..

[cit21] Sengupta A., Sunder A. V., Sohoni S. V., Wangikar P. P. (2018). J. Biotechnol..

[cit22] Köninger K., Gómez-Baraibar Á., Mügge C., Paul C. E., Hollmann F., Nowaczyk M. M., Kourist R. (2016). Angew. Chem., Int. Ed..

[cit23] Barone G. D., Hubáček M., Malihan-Yap L., Grimm H. C., Nikkanen L., Pacheco C. C., Tamagnini P., Allahverdiyeva Y., Kourist R. (2023). Biotechnol. Biofuels Bioprod..

[cit24] Tanaka K., Shimakawa G., Tabata H., Kusama S., Miyake C., Nakanishi S. (2021). Photosynth. Res..

[cit25] Sheng X., Yan M., Xu L., Wei M. (2016). J. Mol. Catal. B: Enzym..

[cit26] Fitzpatrick T. B., Amrhein N., Macheroux P. (2003). J. Biol. Chem..

[cit27] Richter N., Gröger H., Hummel W. (2011). Appl. Microbiol. Biotechnol..

[cit28] Hall M., Stueckler C., Hauer B., Stuermer R., Friedrich T., Breuer M., Kroutil W., Faber K. (2008). Eur. J. Org. Chem..

[cit29] Ribeaucourt D., Höfler G. T., Yemloul M., Bissaro B., Lambert F., Berrin J. G., Lafond M., Paul C. E. (2022). ACS Catal..

[cit30] Nett N., Duewel S., Richter A. A., Hoebenreich S. (2017). ChemBioChem.

[cit31] Nett N., Duewel S., Schmermund L., Benary G. E., Ranaghan K., Mulholland A., Opperman D. J., Hoebenreich S. (2021). Mol. Catal..

[cit32] Tüllinghoff A., Djaya-Mbissam H., Toepel J., Bühler B. (2023). Plant Biotechnol. J..

[cit33] Hobisch M., Spasic J., Malihan-Yap L., Barone G. D., Castiglione K., Tamagnini P., Kara S., Kourist R. (2021). ChemSusChem.

[cit34] Valotta A., Malihan-Yap L., Hinteregger K., Kourist R., Gruber-Woelfler H. (2022). ChemSusChem.

[cit35] Hoschek A., Heuschkel I., Schmid A., Bühler B., Karande R., Bühler K. (2019). Bioresour. Technol..

[cit36] Xu L., Weathers P. J., Xiong X., Liu C. (2009). Eng. Life Sci..

[cit37] Sastre R. R. and Posten C., in Biotechnological Applications with Microalgae, ed. M. Rögner, De Gruyter, 2021, pp. 175–202

[cit38] Behle A., Saake P., Germann A. T., Dienst D., Axmann I. M. (2020). ACS Synth. Biol..

[cit39] Hall M., Stueckler C., Ehammer H., Pointner E., Oberdorfer G., Gruber K., Hauer B., Stuermer R., Kroutil W., Macheroux P., Faber K. (2008). Adv. Synth. Catal..

[cit40] Quilter H. C., Hutchby M., Davidson M. G., Jones M. D. (2017). Polym. Chem..

[cit41] Yakabi K., Mathieux T., Milne K., López-Vidal E. M., Buchard A., Hammond C. (2017). ChemSusChem.

[cit42] Akutagawa S. (1997). Top. Catal..

[cit43] Perin G., Morosinotto T. (2023). Front. Biol..

[cit44] Imada T., Yamamoto C., Toyoshima M., Toya Y., Shimizu H. (2023). Biotechnol. Prog..

[cit45] Xu L., Weathers P. J., Xiong X., Liu C. (2009). Eng. Life Sci..

[cit46] Dong Z., Wen Z., Zhao F., Kuhn S., Noël T. (2021). Chem. Eng. Sci..

[cit47] Sheldon R. A. (2017). Green Chem..

[cit48] Lin B., Tao Y. (2017). Microb. Cell Fact..

[cit49] Woodley J. M. , in Synthetic Methods for Biologically Active Molecules, ed. E. Brenna, Wiley-VCH, 2013, pp. 263–284

[cit50] Katz M., Frejd T., Hahn-Hägerdal B., Gorwa-Grauslund M. F. (2003). Biotechnol. Bioeng..

[cit51] Szczepańska E., Colombo D., Tentori F., Olejniczak T., Brenna E., Monti D., Boratyński F. (2021). Sci. Rep..

[cit52] Fuhrer T., Fischer E., Sauer U. (2005). J. Bacteriol..

[cit53] Ernst M., Kaup B., Müller M., Bringer-Meyer S., Sahm H. (2005). Appl. Microbiol. Biotechnol..

[cit54] Kratzer R., Pukl M., Egger S., Vogl M., Brecker L., Nidetzky B. (2011). Biotechnol. Bioeng..

[cit55] Mähler C., Burger C., Kratzl F., Weuster-Botz D., Castiglione K. (2019). Molecules.

[cit56] Rapp C., Nidetzky B., Kratzer R. (2021). J. Biotechnol..

[cit57] Böhmer S., Köninger K., Gómez-Baraibar Á., Bojarra S., Mügge C., Schmidt S., Nowaczyk M., Kourist R. (2017). Catalysts.

[cit58] McElroy C. R., Constantinou A., Jones L. C., Summerton L., Clark J. H. (2015). Green Chem..

[cit59] Constable D. J. C., Curzons A. D., Cunningham V. L. (2002). Green Chem..

[cit60] Wang D. S., Chen Q.
A., Lu S. M., Zhou Y. G. (2012). Chem. Rev..

[cit61] Winkler C. K., Schrittwieser J. H., Kroutil W. (2021). ACS Cent. Sci..

[cit62] Sheldon R. A. (2023). Green Chem..

[cit63] Brown S. and Jones D., European Electricity Review 2024, London, 2024

[cit64] de María P. D. (2024). RSC Sustainability.

[cit65] Alfaro-Sayes D. A., Amoah J., Aikawa S., Matsuda M., Hasunuma T., Kondo A., Ogino C. (2022). Biochem. Eng. J..

[cit66] Rissanen V., Vajravel S., Kosourov S., Arola S., Kontturi E., Allahverdiyeva Y., Tammelin T. (2021). Green Chem..

[cit67] Hoschek A., Toepel J., Hochkeppel A., Karande R., Bühler B., Schmid A. (2019). Biotechnol. J..

[cit68] Xie H., Kjellström J., Lindblad P. (2023). Biotechnol. Biofuels Bioprod..

[cit69] Miao R., Xie H., Lindblad P. (2018). Biotechnol. Biofuels.

[cit70] Kukil K., Lindberg P. (2024). Microb. Cell Fact..

[cit71] Rueda E., Altamira-Algarra B., García J. (2022). Bioresour. Technol..

[cit72] Veetil V. P., Angermayr S. A., Hellingwerf K. J. (2017). Microb. Cell Fact..

[cit73] Matsudaira A., Hoshino Y., Uesaka K., Takatani N., Omata T., Usuda Y. (2020). J. Biosci. Bioeng..

[cit74] Rodrigues J. S., Lindberg P. (2021). Metab. Eng. Commun..

[cit75] Touloupakis E., Rontogiannis G., Benavides A. M. S., Cicchi B., Ghanotakis D. F., Torzillo G. (2016). Int. J. Hydrogen Energy.

[cit76] Gao Z., Zhao H., Li Z., Tan X., Lu X. (2012). Energy Environ. Sci..

[cit77] Liu X., Miao R., Lindberg P., Lindblad P. (2019). Energy Environ. Sci..

[cit78] Tieves F., Tonin F., Fernández-Fueyo E., Robbins J. M., Bommarius B., Bommarius A. S., Alcalde M., Hollmann F. (2019). Tetrahedron.

[cit79] Włodarczyk A., Selão T. T., Norling B., Nixon P. J. (2020). Commun. Biol..

[cit80] Li C., Wen A., Shen B., Lu J., Huang Y., Chang Y. (2011). BMC Biotechnol..

[cit81] Özgen F. F., Runda M. E., Burek B. O., Wied P., Bloh J. Z., Kourist R., Schmidt S. (2020). Angew. Chem., Int. Ed..

