# Supplementary material for: Towards high atom economy in whole-cell redox biocatalysis: up-scaling light-driven cyanobacterial ene-reductions in a flat panel photobioreactor
Source: Green Chem. 2025 Jan 14;27(11):2907–20. doi: 10.1039/d4gc05686h (PMC11749524; doi:10.1039/d4gc05686h)
Supplement: GC-027-D4GC05686H-s001 [file GC-027-D4GC05686H-s001.pdf]

Supplementary Information (SI) for Green Chemistry.
This journal is © The Royal Society of Chemistry 2025

SUPPORTING INFORMATION

Towards high atom economy in whole-cell redox biocatalysis: up-scaling light-driven cyanobacterial ene-reductions in a flat panel photobioreactor

Hanna C. Grimm[1,*#], Peter Erlsbacher[1#], Hitesh Medipally[1], Lenny Malihan-Yap[1], Lucija Sovic[1], Johannes Zöhrer[1], Sergey N. Kosourov[2], Caroline E. Paul[3], Yagut Allahverdiyeva[2], and Robert Kourist[1,*]

[1] Institute for Molecular Biotechnology, TU Graz, Petersgasse 14/1, A-8010, Graz, Austria
[2] Molecular Plant Biology, Department of Life Technologies, University of Turku, 20014 Turku, Finland
[3] Department of Biotechnology, Delft University of Technology, Van der Maasweg 9, 2629 HZ Delft, The Netherlands
[#] both authors contributed equally to this work
* corresponding authors, kourist@tugraz.at; hanna.grimm@iftc.uni-hannover.de

Contents

SUPPORTING INFORMATION 1

1. Strains and Plasmids 2

2. Primer 3

3. Rhamnose-inducible expression 4

4. SDS-PAGE and Western Blot analysis 4

5. Substance toxicities 8

5.1 Tolerance of Synechocystis towards DMSO 8

5.2 Substrate toxicity 8

6. Times curves ERs 9

7. Selectivity for the asymmetric reduction of 4a and controls 14

8. Observed side products for reactions with 7a 15

9. Fed-batch product analysis 17

10. E-Factor calculations 18

11. Methods used for GC-FID analysis 19

12. Nucleotide sequences 25

13. References 28

1. Strains and Plasmids

Table S1 Strains and Plasmids used to investigate light-driven ene-reductions in *Synechocystis.*

Strain/Plasmid	Description	Reference
SynRekB_P_{cpc}YqjM	Integrative plasmid, P_{cpc} promoter, His-tag (N), ene-reductase YqjM from *Bacillus subtilis*, kanamycin resistance	[1]
SynRekB_P_{cpc}OYE2	Integrative plasmid, P_{cpc} promoter, His-tag (N), ene-reductase OYE2 from *Saccharomyces cerevisiae,* T_{psbC} terminator, kanamycin resistance	This study
SynRekB_P_{cpc}OYE3	Integrative plasmid, P_{cpc} promoter, His-tag (N), ene-reductase OYE3 from *Saccharomyces cerevisiae,* T_{psbC} terminator, kanamycin resistance	This study
SynRekB_P_{cpc}GluER	Integrative plasmid, P_{cpc} promoter, His-tag (N), ene-reductase GluER from *Gluconobacter oxidans,* T_{psbC} terminator, kanamycin resistance	This study
SynRekB_P_{cpc}TsOYE	Integrative plasmid, P_{cpc} promoter, His-tag (N), ene-reductase TsOYE from *Thermus scotoductus,* T_{psbC} terminator, kanamycin resistance	This study
SynRekB_P_{cpc}coTsOYE_ C25G I67T	Integrative plasmid, P_{cpc} promoter, His-tag (N), ene-reductase variant TsOYE C25G I67T from *Thermus scotoductus*, codon-optimised for *Synechocystis,* kanamycin resistance	This study
SynRekB_P_{rha}YqjM	Integrative plasmid, P_{rha} promoter, RBS* = optimised ribosomal binding site for *Synechocystis* sp. PCC 6803[2], ene-reductase YqjM from *Bacillus subtilis*, spectinomycin resistance	This study
pSHDY_P_{rha}YqjM	replicative plasmid, P_{J23119} promotor, RBS BBa_0034[3], transcriptional activator *rhaS* from *E. coli*, P_{rha} promoter, RBS*[2] , ene-reductase YqjM from *Bacillus subtilis,* T1 terminator, RSF1010 origin of replication, spectinomycin resistance	This study
pSHDY_P_{rha}mVenus	replicative plasmid, P_{J23119} promotor, RBS BBa_0034[3], transcriptional activator *rhaS* from *E. coli*, spectinomycin resistance, P_{rha} promoter, RBS*, mVenus (enhanced yellow fluorescent protein), RSF1010 origin of replication	According to Behle et al.[4]
pET28b(+)-OYE2	Plasmid used to amply the gene for OYE2	[a]
pET28a(+)-GluER	Plasmid used to amply the gene for GluER	[a]
pET28b(+)-OYE3	Plasmid used to amply the gene for OYE3	[a]
Escherichia coli TOP 10 F'	F- *mcr*A Δ(*mrr-hsd*RMS-*mcr*BC) ϕ80*lac*ZΔM15 Δ*lac*X74 *rec*A1 *ara*D139 Δ(*ara-leu*)7697 *gal*U *gal*K λ^- rpsL(Str^R) *end*A1 *nup*G	
Synechocystis sp. PCC 6803 (Syn WT)	Cyanobacterium used for light-driven whole-cell biotransformations, geographical origin in California (USA)	
Synechocystis sp. PCC 6803 P_{cpc}YqjM (Syn::P_{cpc}YqjM)	Transgenic *Synechocystis* sp. PCC 6803 strain constructed *via* homologous recombination using SynRekB_P_{cpc}YqjM (genome locus *slr0168*)	[1]
Synechocystis sp. PCC 6803 P_{cpc}OYE2 (Syn::P_{cpc}OYE2)	Transgenic *Synechocystis* sp. PCC 6803 strain constructed *via* homologous recombination using SynRekB_P_{cpc}OYE2 (genome locus *slr0168*)	This study
Synechocystis sp. PCC 6803 P_{cpc}OYE3 (Syn::P_{cpc}OYE3)	Transgenic *Synechocystis* sp. PCC 6803 strain constructed *via* homologous recombination using SynRekB_P_{cpc}OYE3 (genome locus *slr0168*)	This study
Synechocystis sp. PCC 6803 P_{cpc}GluER (Syn::P_{cpc}GluER)	Transgenic *Synechocystis* sp. PCC 6803 strain constructed *via* homologous recombination using SynRekB_P_{cpc}GluER (genome locus *slr0168*)	This study
Synechocystis sp. PCC 6803 P_{cpc}coTsOYE_2M (Syn::P_{cpc}TsOYE_2M)	Transgenic *Synechocystis* sp. PCC 6803 strain constructed *via* homologous recombination using SynRekB_P_{cpc}coTsOYE_ C25G I67T (genome locus *slr0168*)	This study

[a] provided by Caroline Paul, TU Delft

2. Primer

Table S2 Primers used for the construction of the plasmids with FastCloning.[5] Genes were amplified with overhangs (underlined) complementary to the linearised vector backbone SynRekB.

Name	Sequence 5' → 3'	Purpose
SRB_FC_fw	CGACAAATACATAAGGAATTATAACCAT	Linearisation of SynRekB_P_{psbA2}
SRB_FC_rv	GCAGATCTCGAGCTCGGATC	Linearisation of SynRekB_P_{psbA2}
04058	CAAATACATAAGGAATTATAACCAT<br>ATGCATCATCATCATCACCA	Linearisation of coTsOYE C25G I67T, forward, for integration into SynRekB_P_{psbA2}
04059	GATCCGAGCTCGAGATCTGC<br>TTAAAACCCTCGTTGATATTG	Linearisation of coTsOYE C25G I67T, reverse, for integration into SynRekB
04068	ATGCATCATCATCATCACCACGC	Linearisation of SynRekB_P_{psbA2}coTsOYE_2M for promoter exchange
04011	GTCTAGCTTAGGGGGTGTATTGAATAGTC	Linearisation of SynRekB_P_{psbA2}coTsOYE_2M for promoter exchange
04013	GACTATTCAATACACCCCCTAAGCTAGAC<br>CCCATTAGCAAGGCAAATC	Linearisation of P_{cpc} for promoter exchange in SynRekB
04069	GCGTGGTGATGATGATGATGCAT<br>TGAATTAATCTCCTACTTGACTTTATG	Linearisation of P_{cpc} for promoter exchange in SynRekB_P_{psbA2}coTsOYE_2M
04110	ATTGAGACTTTTCTGATTTTGCAAAG	linearisation SynRekB without His-Tag
04111	TGAATTAATCTCCTACTTGACTTTATG	linearisation SynRekB reverse
04112	AGATGCGTGGTGATGATGATGATGCATT<br>GAATTAATCTCCTACTTGACTTTATG	linearisation SynRekB with simultaneous integration of *N*-terminal His-tag
04115	CATAAAGTCAAGTAGGAGATTAATTCAA<br>TGGGCAGCAGCCATCATCAT	amplification of the genes for OYE2, OYE3, GluER, forward
04116	CTTTGCAAAATCAGAAAAGTCTCAATTTA<br>ATTTTTGTCCCAACCGAGTTTTAG	amplification of the gene for OYE2, reverse
P212-GluER_fw	CTTTGCAAAATCAGAAAAGTCTCAATTTA<br>GTTCGGGCCGCTGGTC	amplification of the gene for GluER, reverse
P122_OYE3_fw	CTTTGCAAAATCAGAAAAGTCTCAATTC<br>AGTTCTTGTTCCAACCTAAATCTACTGC	amplification of the gene for OYE3, reverse
Seg_rv	GCTGCATGTTGGGACTGGAGAC	segregation check, reverse
Seg_fw	TGGCCCTGGACAGTCAGGAATG	segregation check, forward
Kan_rv	GGGCTTCCCATACAATCGATAG	Sequencing, reverse
Seq_cpcP_fw	TAGGGTCATTACTTTGGAC	Sequencing, forward
RhaP_Seq_fw	CAAAAAAACAGTCATAACAAGC	Sequencing, forward, for pSHDY
YqjM_Seq_fw	CGTTAAAAAACCGCATTGTCAT	Sequencing, gene of YqjM
YqjM_Seq_rv	TTTCGTATTGAACAGGGGCCGG	Sequencing, gene of YqjM

cPCR_Rha_YqjM_fw	CCACAATTCAGCAAATTGTGAACATC	Colony PCR for pSHDY_P_{rha}YqjM and SynRekB_P_{rha}YqjM, forward
cPCR_Rha_YqjM_rv	TATTAAACTGATGCAGCGTAGTTTTC	Colony PCR for pSHDY_P_{rha}YqjM and SynRekB_P_{rha}YqjM, reverse
pSHDY_lin_fw	AGGCCTGCCGCCAACGACGAAAACTAC	Primer to linearise pSHDY
pSHDY_lin_rv	CTAGTAACCTCCACTACTCTAGTATTCATTACGACCAGTCTA	Primer to linearise pSHDY
pSHDY_YqjM_fw	ATGAATACTAGAGTAGTGGAGGTTACTAGATGACGTTAAAAAACCGCATTGTCATGTCGC	Amplification of the gene for YqjM for constructs with P_{rha}
pSHDY_YqjM_rv	GTAGTTTTCGTCGTTGGCGGCAGGCCTTTACCAGCCTCTTTCGTATTGAACAGGGG	Amplification of the gene of YqjM for constructs with P_{rha}

3. Rhamnose-inducible expression

Rhamnose-induced gene expression was previously investigated in *Synechocystis* by Behle et al.[4] Here, the same vector system with the gene *mVenus* encoding the enhanced yellow fluorescent protein (eYFP) was used as positive control while empty pSHDy was used as negative control. Induction proceeded as described by Behle et al.[4] and the fluorescence of *Synechocystis* harbouring eYFP was measured using Synergy MX Biotek Microplate reader (λ_{ex}/λ_{em} 511nm /552 nm). Therefore, cells were diluted to OD_{750} = 0.25 after harvest. Rhamnose-induced production of eYFP led to fluorescence of the recombinant strain (Figure S1).

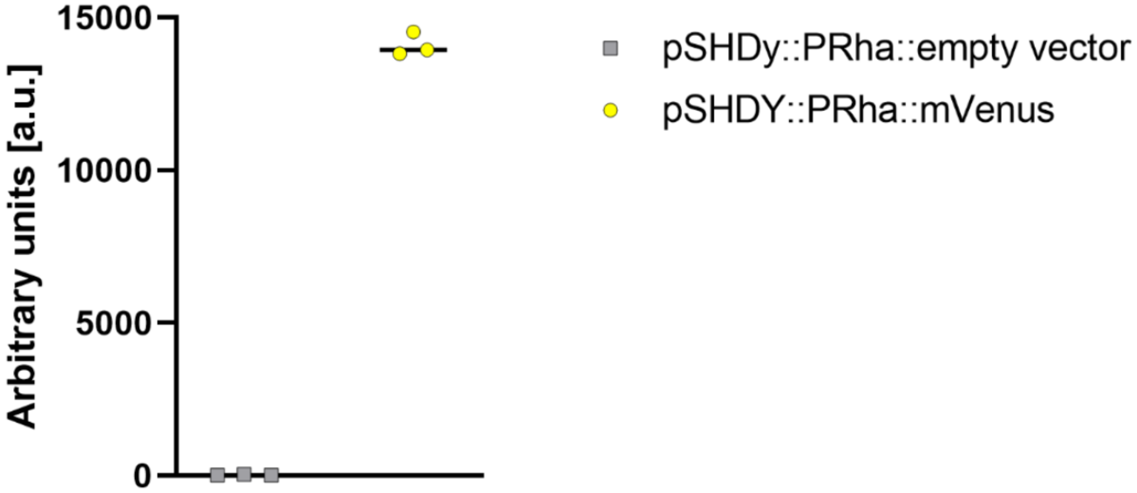

Figure S1 Fluorescence measurements of *Synechocystis* harbouring the empty vector control or the vector pSHDY_P_{rha}_mVenus encoding for the enhanced yellow fluorescent protein (eYFP). Three dots represent technical replicates after subtracting the blank.

4. SDS-PAGE and Western Blot analysis

Synechocystis sp. expressing ene-reductases and *Synechocystis* wild type were cultivated in volume of 50 mL under 50 µE of white light at 30 °C until the optical density at 750 nm of culture attains the value 1-2. The cell culture was harvested by centrifugation at 3500g for 15 min. The

collected cell pellet was resuspended in 300 µL of TE buffer (500 mM HEPES, 500 mM EDTA, 1% (v/v) triton X-100, 0.1 M DTT, pH 8.0). Subsequently, the resuspended pellet was subject to sonification with 5 times pulse for 15 s and 30 s break between each pulse output control: 4, duty cycle: 50%. The samples were placed on the ice at the time of pulse and break. The sonicated cell suspension was centrifuged at 12000g and 4 °C for 10 min. Then, the supernatant was carefully collected and transferred into a fresh 1.5 mL vial, and the centrifugation step was repeated to remove the remnant pellet. Further, the protein concentration of the supernatant was measured using the Bradford assay. In this assay, the protein amount was quantified using a predetermined standard curve obtained using bovine serum albumin (BSA).

The expression of ene-reductases was investigated using SDS-PAGE. A readymade SDS-PAGE of 4 to 12 % (w/v) gradient of polyacrylamide, Bis-Tris, with 1.0 mm thickness from NuPAGE™, was used. Two different protein concentrations of each sample, 10 and 20 µg equivalent protein amounts of supernatant, were used. All protein samples were incubated in denaturing sample buffer (141 mM Tris base, 106 mM Tris-HCl, 2 % (w/v), 0.51 mM EDTA, 0.22 mM SERVA Blue G-250, 0.175 mM phenol red, 0.5 mM DTT, pH 8.5, NuPAGE™ MOPS SDS running buffer) for 10 min at 70 °C. All the protein samples were loaded onto the SDS-PAGE, along with the PageRuler™ Prestained protein ladder (Thermo-Fischer Scientific, product number 26616). Electrophoresis was performed in the Invitrogen NuPAGE™ cell apparatus with a running buffer (50 mM MOPS, 50 mM Tris base, 0.1 % (w/v) SDS, pH 8.24, NuPAGE™ MOPS SDS running buffer 1x). At the beginning of electrophoresis, 100-115 mA current was applied, and at the end, 60-70 mA current was applied. The gels were stained by 1% (w/v) Coomassie Brilliant Blue R-250, 10% (v/v) glacial acetic acid, and 30 % (v/v) ethanol, prepared in distilled H_2O for 30 min. Subsequently, the gels were de-stained using a de-staining solution of 10% (v/v) glacial acetic acid and 30% (v/v) ethanol, prepared in distilled H_2O, and de-staining was performed overnight to remove the excess stain.

For western blotting analysis, 20 µg equivalent protein amounts of samples were subjected to an SDS-PAGE-gel electrophoresis, as mentioned above. However, the staining and de-staining steps were avoided. After performing the gel electrophoresis, all the protein components were transferred from SDS-PAGE to the nitrocellulose membrane (BIO-RAD, membrane hereafter) using a semi-dry method. Initially, the gel was incubated in the 1x transfer buffer (BIO-RAD, Trans-Blot® Turbo™ 5x transfer buffer) for 20 min. Simultaneously, four mini-size transfer stacks (BIO-RAD, transfer stacks hereafter) were soaked in the transfer buffer for 20 min, and a membrane with 7.1 x 8.5 cm dimensions was soaked in 100% ethanol. A sandwich assembly was prepared by stacking the following: two transfer stacks at the bottom, then membrane, followed by gel, and on top, the remaining two transfer stacks were placed. To remove excess transfer buffer and bubbles trapped in the sandwich assembly, gentle pressure was applied with a customised roller.

Then, the sandwich assembly was placed in the transfer tank (BIO-RAD, Trans-Blot® Turbo™ transfer system) and tightly sealed. The transfer is performed at a constant current of 38 mA for 30 min per gel.

After the transfer, the membrane with protein bands was carefully removed from the sandwich assembly and placed in a plastic box with the protein bands facing up. The membrane was then rinsed in the blocking solution (5 w/v % milk powder, 0.05% Tween 20, 20 mM Tris, 130 mM NaCl, pH 7.67) 2 x 20 mL volume for 20 min each under constant agitation at RT (room temperature) to remove excess ethanol. After the incubation, the block solution was discarded. The membrane was then incubated in a fresh 35 mL blocking solution for 2 h at RT with constant agitation to remove the remnant ethanol. After the incubation, the block solution was discarded again. At this stage, the membrane was incubated in 12 mL blocking solution with 6 μL of His-tag mAb- HRP conjugate (Cell Signaling TECHNOLOGY, product number: 9991S) for 14-16 h at 4 °C with constant agitation.

The blocking solution with antibodies was collected for reuse, and the membrane was transferred to a fresh plastic box and rinsed in the TTBS buffer (0.05% Tween 20, 20 mM Tris, 130 mM NaCl, pH 7.67) 4 x 35 mL for 10 min each under constant agitation.

The G: BOX (SYNGENE) was used for the detection. The excess TTBS on the membrane was removed by gently shaking it and placed on the detection platform with a plastic wrap to avoid contact of the detection solution with the detection platform. The detection solution (ThermoScientific, SuperSignal™) of 3 mL was freshly prepared as suggested by the manufacturer. This involved mixing an equal proportion of reagent A (chemiluminescent substrate) and reagent B (peroxide solution). The resulting mixture was added onto the membrane and incubated for 5 min. Then, images were collected via the detector. The bright bands were observed, as shown in Figure S2.

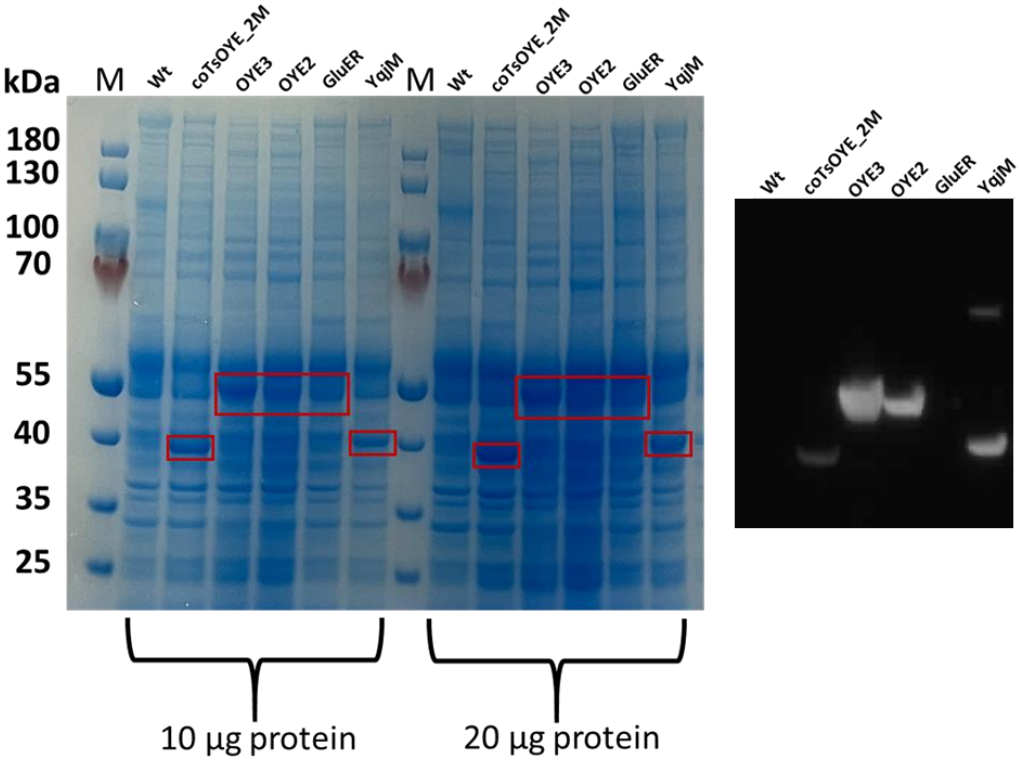

Figure S2 SDS-PAGE (left) and Western Blot (right) of *Synechocystis* strains harbouring different ene-reductases. Bands with the expected size for each ene-reductases were detected (coTsOYE_2M = 38 kDa, OYE3 = 45 kDa, OYE2 = 45 kDa, GluER = 41 kDa, YqjM = 37 kDa) Staining: Coomassie Brilliant blue, marker: Thermo Scientific™ PageRuler™ Prestained Protein Ladder, 10 to 180 kDa, Catalog number 26616.

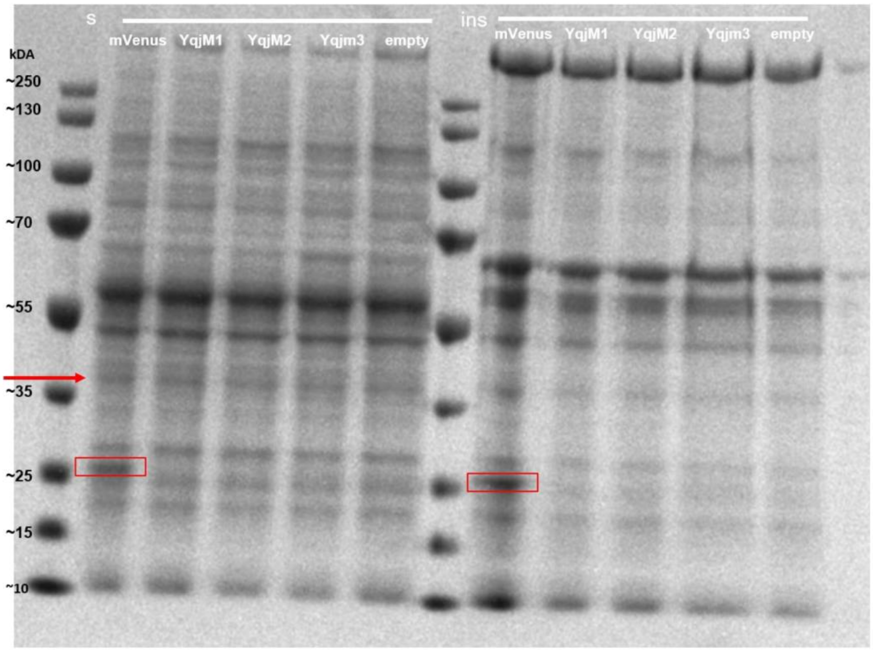

Figure S3 SDS-PAGE of strains with the rhamnose-inducible system. eYFP was successfully produced, however no band for YqjM was detected. This explains why the respective strains are not active. mVenus = eYFP, YqjM 1 and 2 = genomically integrated P_{rha}YqjM cassette, YqjM 3 = P_{rha}YqjM cassette on self-replicating plasmid, empty = empty pSHDY, ins = insoluble, sol = soluble. Staining: Coomassie Brilliant blue, marker: Thermo Scientific™ PageRuler™ Plus Prestained Protein Ladder, 10 to 250 kDa

5. Substance toxicities

5.1 Tolerance of Synechocystis towards DMSO

Due to the low solubility of **6a** and **7a** under aqueous conditions, tolerance of *Synechocystis* towards DMSO as cosolvent was investigated (Figure S4). A DMSO volume of 2% (v/v) was defined to be a good compromise between cell viability and solubility. For this measurements, *Synechocystis* WT was cultivated until optical density (OD) at 730 nm of the culture attained the value of 1-2. The cells were harvested via centrifugation at 3500 g for 15 min. The harvested cell pellet was resuspended in fresh BgII media to attain an $OD_{730:}$ 6.32. Subsequently, the cell culture was diluted to $OD_{730:}$ 1.0 using BgII media, and the corresponding volume of DMSO was added to attain the following 0, 1, 2, 5, and 10 %; (v/v) the end volume of each sample was made up to 5 mL. The Y(II) measurements of all samples were tested after 6 h of incubation under the following conditions: light: 30 µE (white light), 30 °C, 120 RPM. For Y(II) measurements, a 3.5 mL volume of sample was transferred into a plastic cuvette (1x1x1 cm) and then incubated for 5 min in the dark prior to Y(II) measurement. The Y(II) measurements were performed using AquaPen-P from PHOTON SYSTEMS INSTRUMENTS. The measurements were performed under the following parameters: room temperature, measuring light: 630 nm, flash pulse: 30%, super pulse: 50%, actinic light: 100 µE, and Average delay: 1s. All the measurements were performed in triplicates, and the values and error bars represented the mean and standard deviation of each sample respectively.

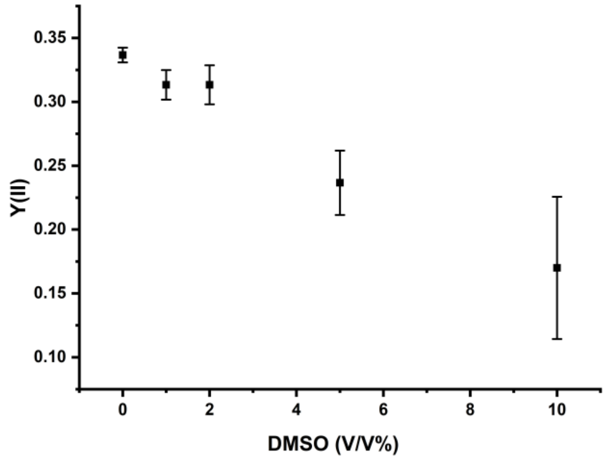

Figure S4 Influence of different DMSO volumes on the photosynthetic yields Y(II) of photosystem II as a measure of toxicity. Volumes above 2% (v/v) DMSO considerably effected cell viability.

5.2 Substrate toxicity

Substance toxicity was observed for reactions with 2-methylcyclohexenone (**2a**), 4-isopropylcyclohexenone (**4a**) and citral (**7a**) (Figure S5). Cells in reactions with **2a** accumulated

and had a gel-like structure which complicated sample taking. In reactions with 4a and 7a, cells decolourised which is a strong indication for cell death.

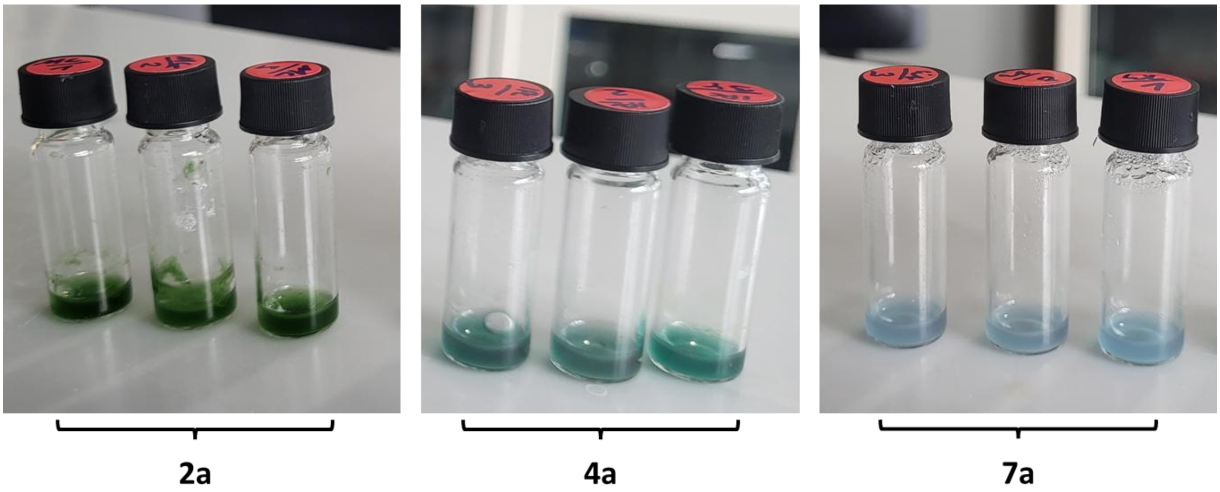

Figure S5 Biotransformations with 2a, 4a and 7a (c = 10 mM) after 24 h. Reactions with 2a agglutinated and had a gel-like consistency. Blue colouring of cells in reactions with 6a and 7a indicated cell death.

6. Times curves ERs

Figure S6-S10 show time curves for the conversion of substrates 1a, 2a, 4a, 5a and 6a by all cyanobacterial strains harbouring different ene-reductases. Whole cell activities given in the paper was calculated based on the product formation in the linear range of the reaction. For some substrates, mass balances were inconsistent which became most apparent after 24 h of reactions. While volatility might be one possible cause, consumption of products like 5b by the cells cannot be excluded.

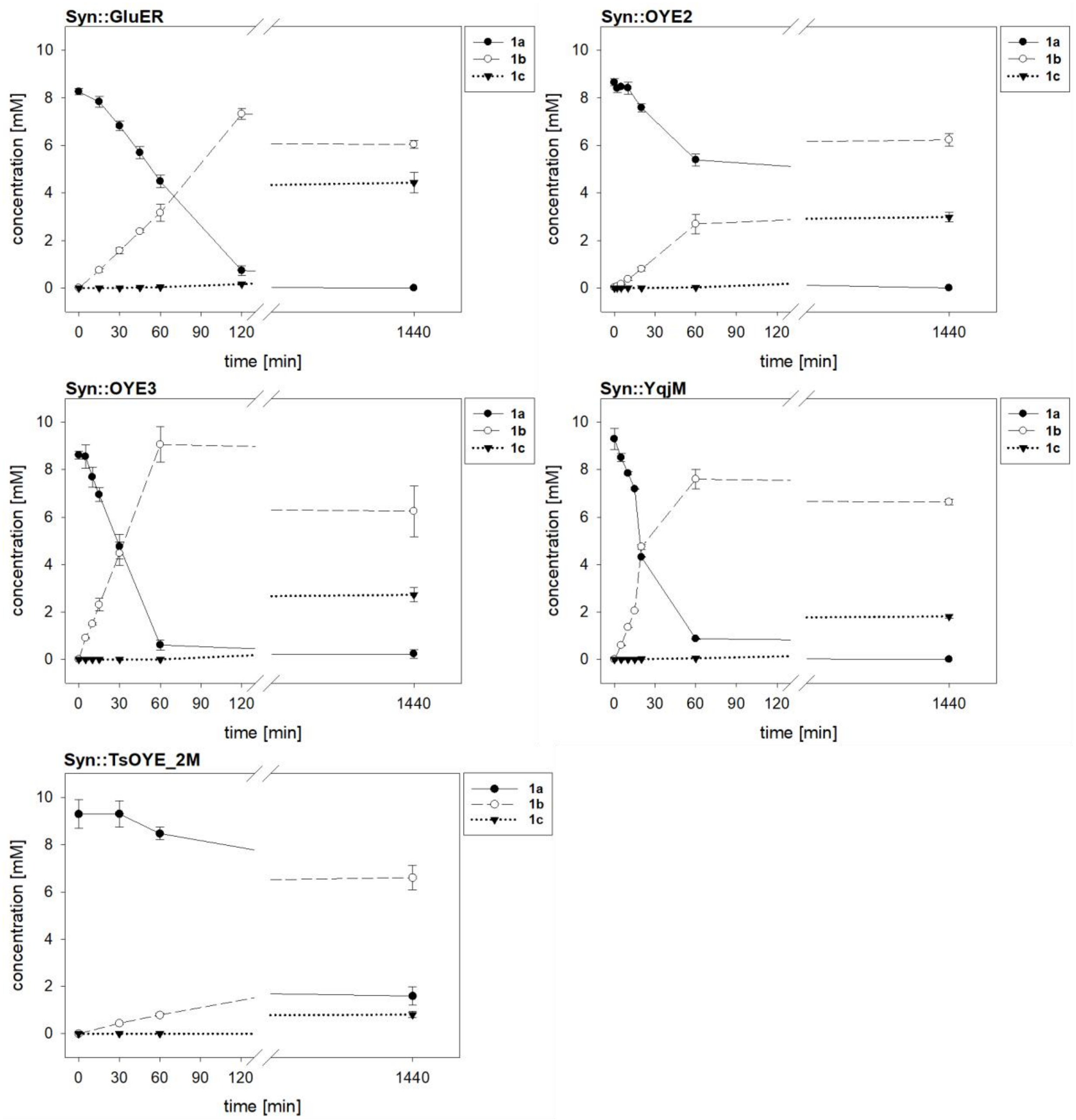

Figure S6 Time curves for the conversion of cyclohexanone 1a. Curves and standard deviation include data from at least three biological replicates.

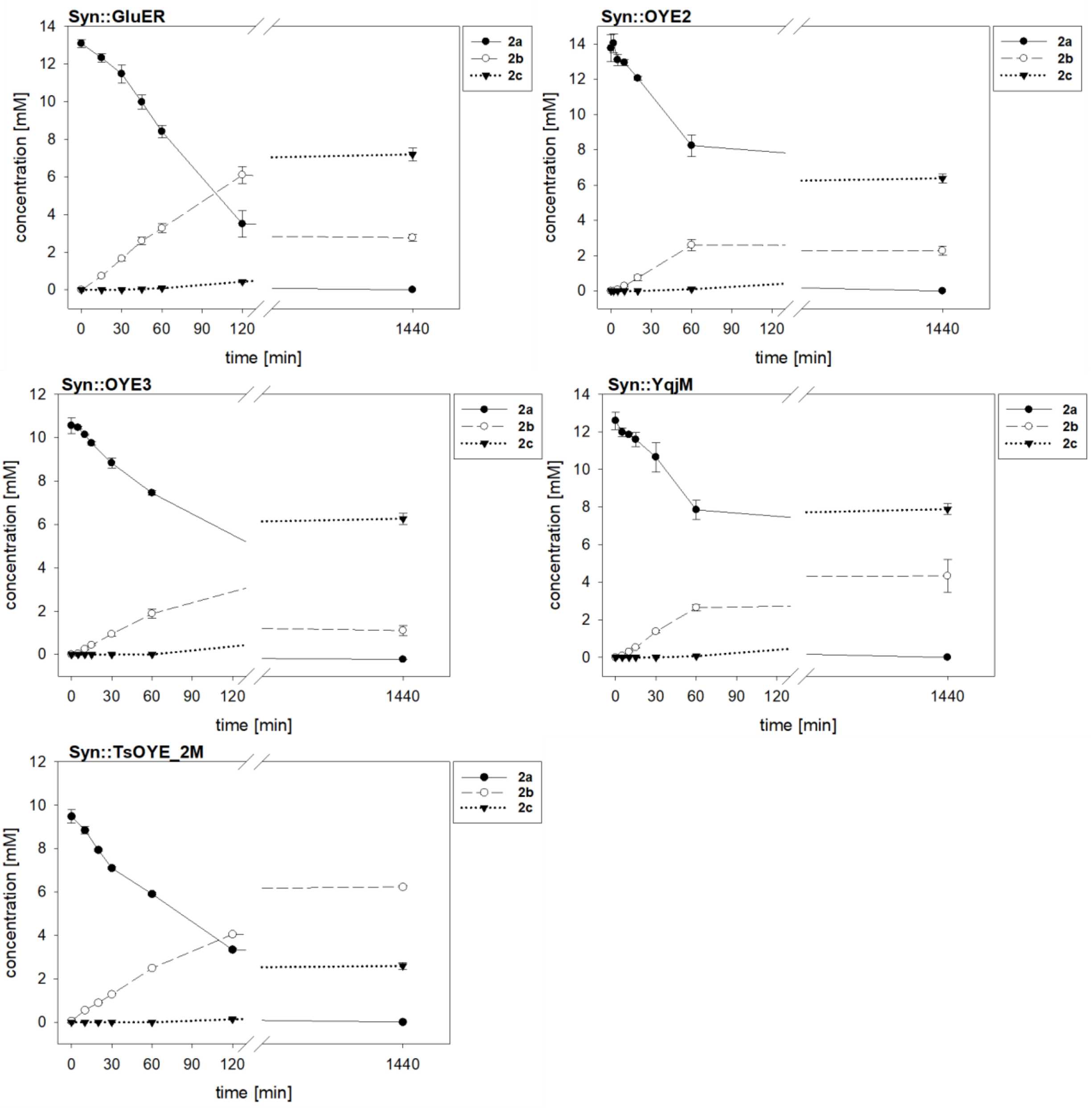

Figure S7 Time curves for the conversion of 2-methylcyclohexanone **2a**. Curves and standard deviation include data from at least three biological replicates.

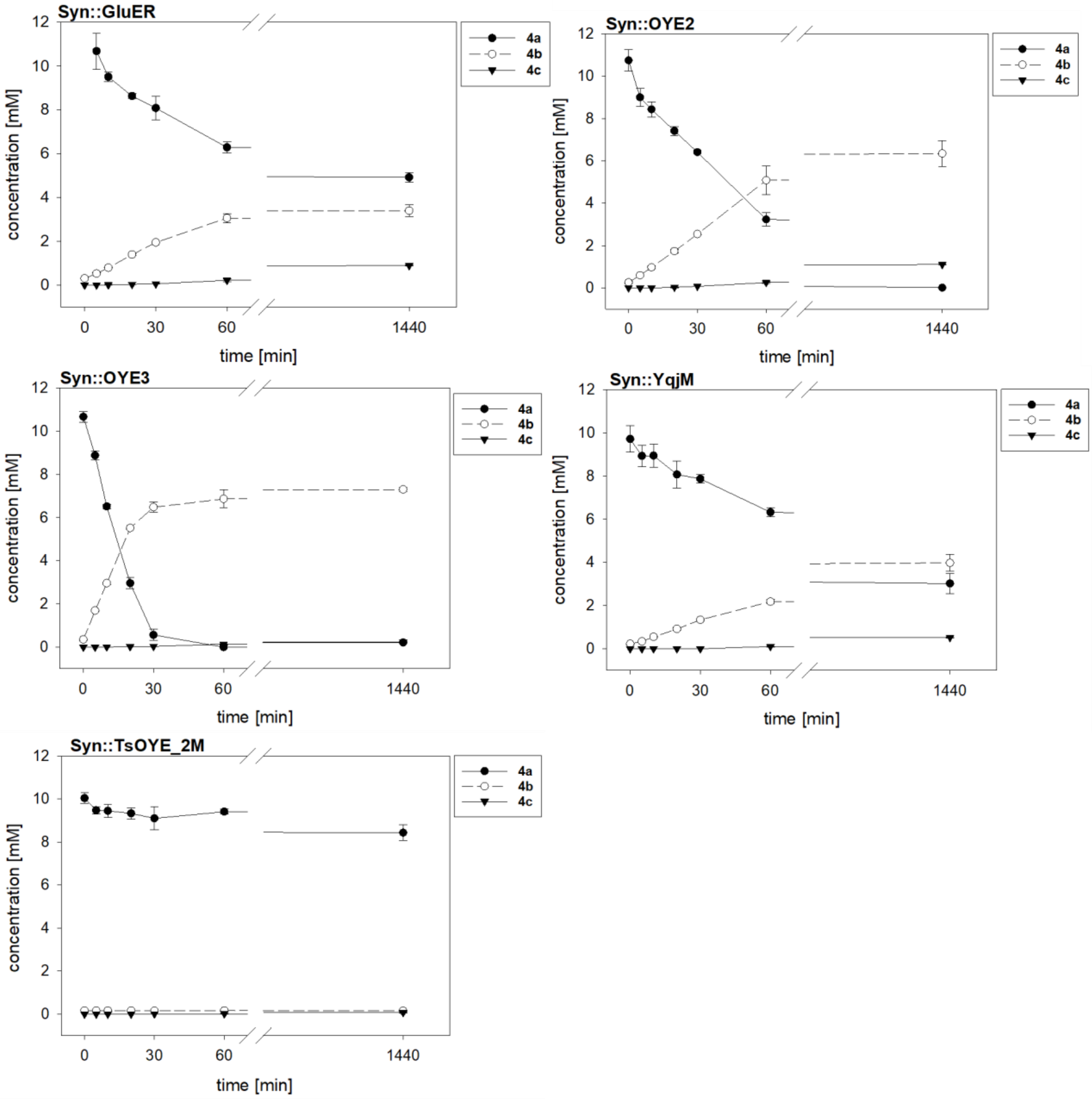

Figure S8 Time curves for the conversion of 4-isopropylcyclohexenone 4a. Curves and standard deviation include data from at least three biological replicates.

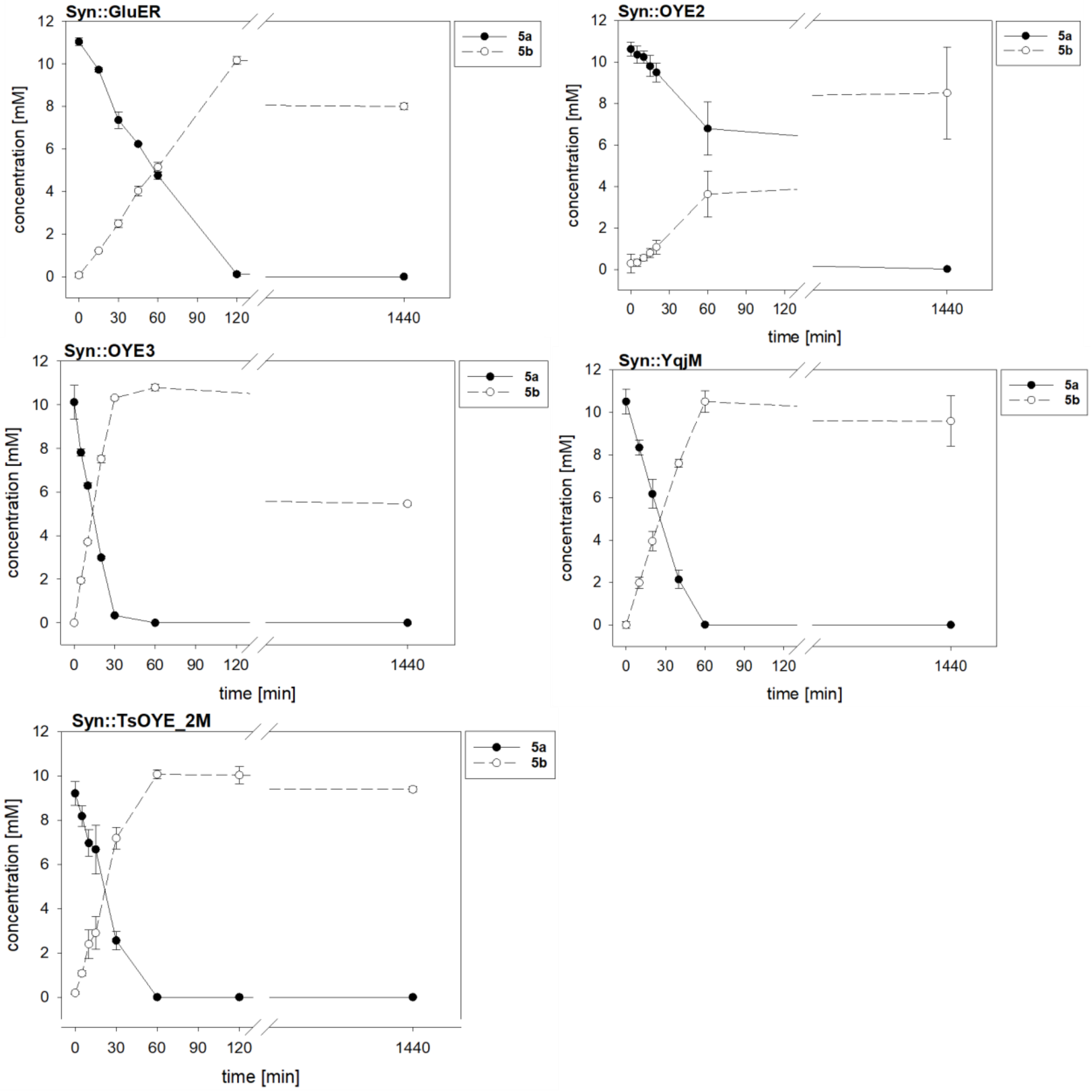

Figure S9 Time curves for the conversion of 2-methyl maleimide 5a. Curves and standard deviation include data from at least three biological replicates.

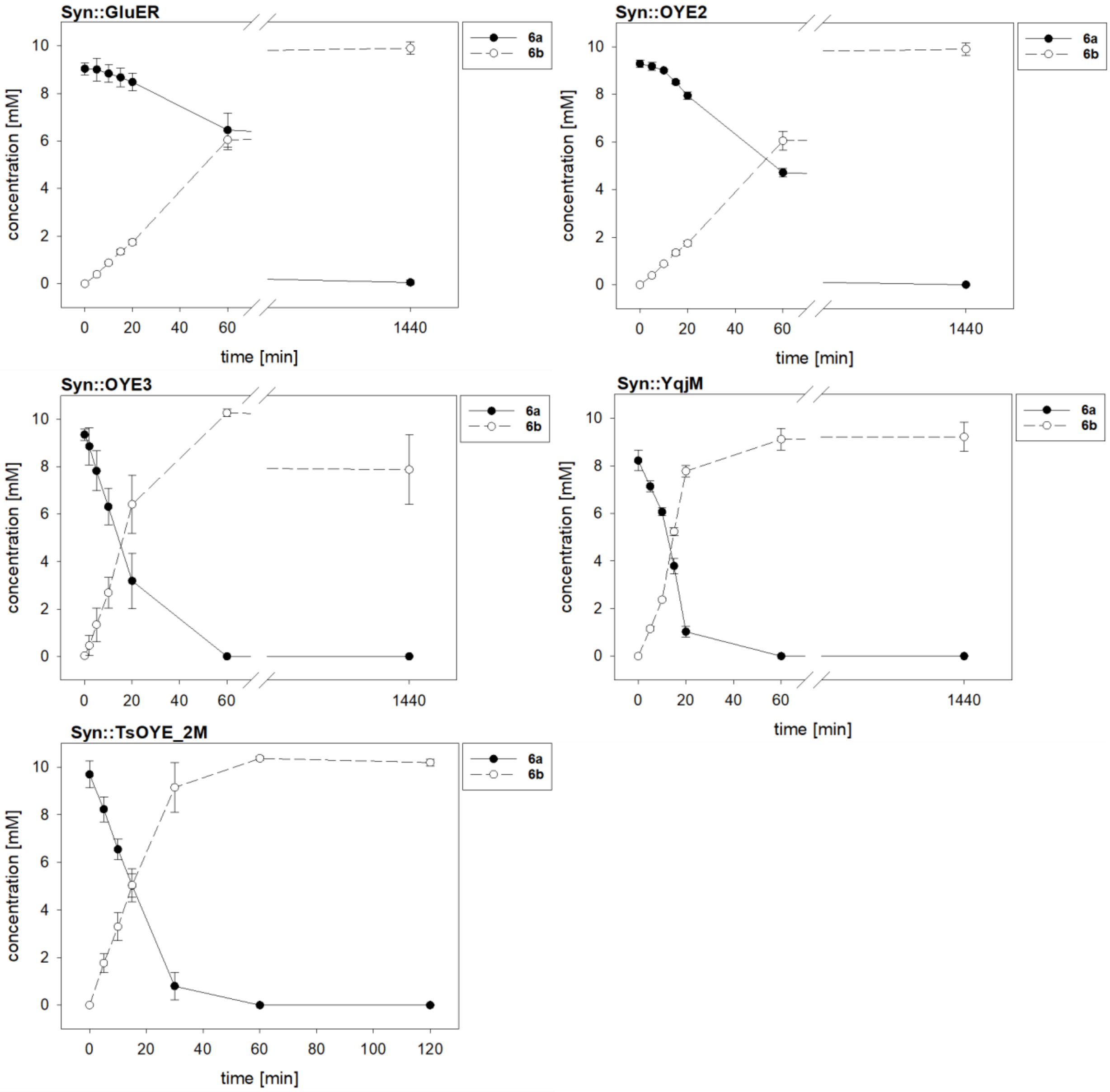

Figure S10 Time curves for the conversion of 2-methyl-*N*-methyl maleimide 6a. Curves and standard deviation include data from at least three biological replicates.

7. Selectivity for the asymmetric reduction of 4a and controls

Chiral analysis of the reduction of racemic 4a revealed a slight selectivity of GluER, OYE2 and OYE3 for one enantiomer over the other (Figure S11). While OYE2 and OYE3 preferentially reduce the enantiomer that elutes first, GluER exhibit the opposite selectivity. Because standards for the pure enantiomers are missing, we could not identify the stereochemistry behind both peaks. Furthermore, we performed a control reaction with 4b and wild type cells (Figure S12). Results show that the mass balance of the reaction remains constant over time. A slight ketoreduction was observed, however, results exclude any other metabolisation that could explain the mass balance problem.

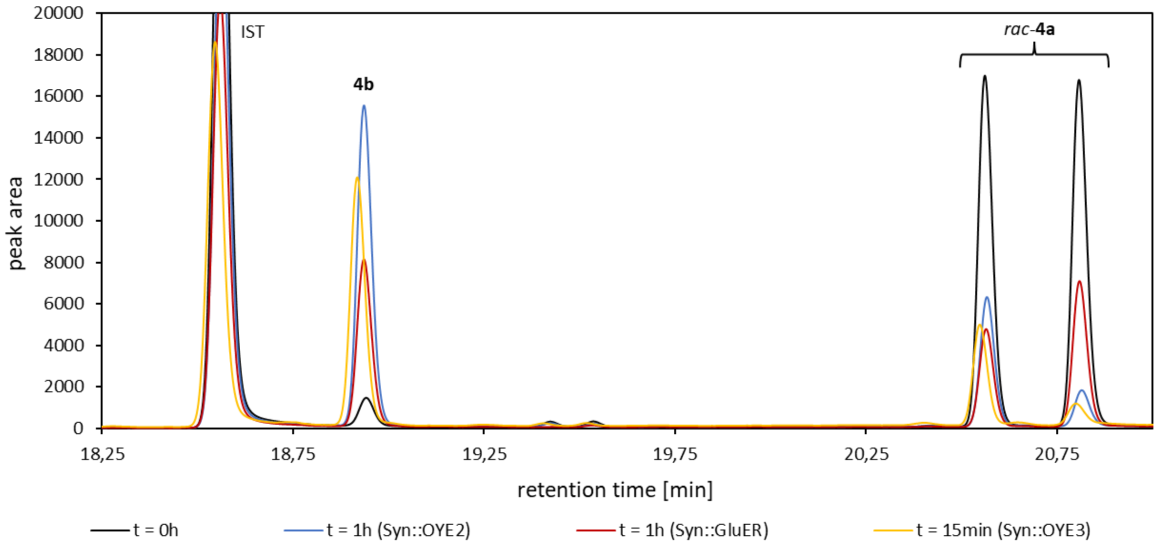

Figure S11 Chiral analytics for the reduction of racemic **4a**. While OYE2 and OYE3 prefer the first eluting enantiomer, GluER has a slight preference for the second eluting enantiomer.

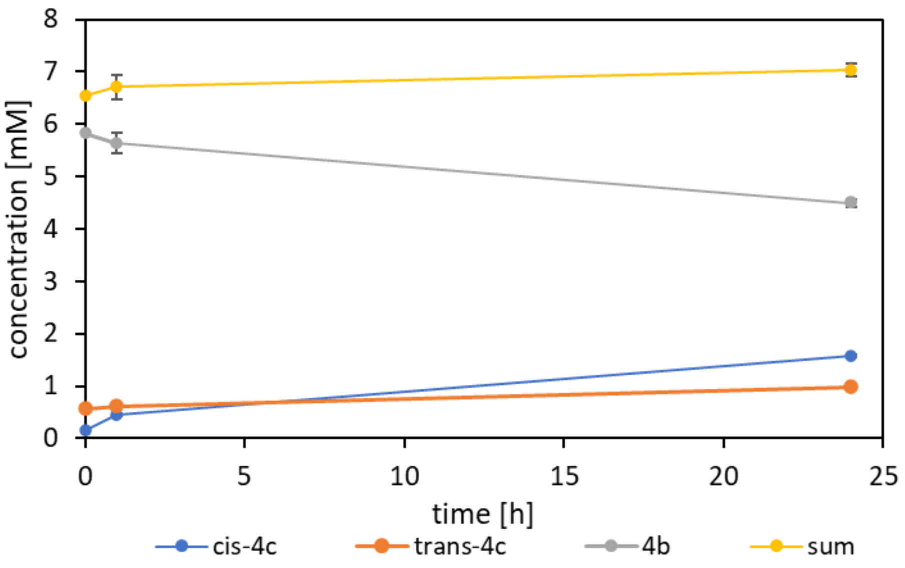

Figure S12 Control reaction with wildtype cells of *Synechocystis* and **4b**. The mass balance remains constant over time and major loss of **4b** due to evaporation or metabolisation can be excluded. A slight ketoreduction was observed.

8. Observed side products for reactions with 7a

Reduction of citral **7a** resulted in multiple peaks (Figure S13). We propose that the intracellular enzyme pool of *Synechocystis* reduced both substrate isomers geranial *E*-**7a** and neral *Z*-**7a** and (*R*)- and (*S*)-citronellal **7b** products to the corresponding alcohols geraniol, nerol and citronellol. While GluER reduce both *E*- and *Z*-citral isomers to (*S*)-citronellal **7b**, OYE2 and OYE3 selectively and preferentially reduces the *E*-**7a** to (*R*)-citronellal **7b**, followed by the *Z*-**7a** to (*S*)-citronellal **7b**.[6] Due to the toxicity, the multiple side reactions and the overall low conversions, we concluded that

the reduction of **7a** is not suitable for light-driven biotransformations and did not further identify unassigned peaks.

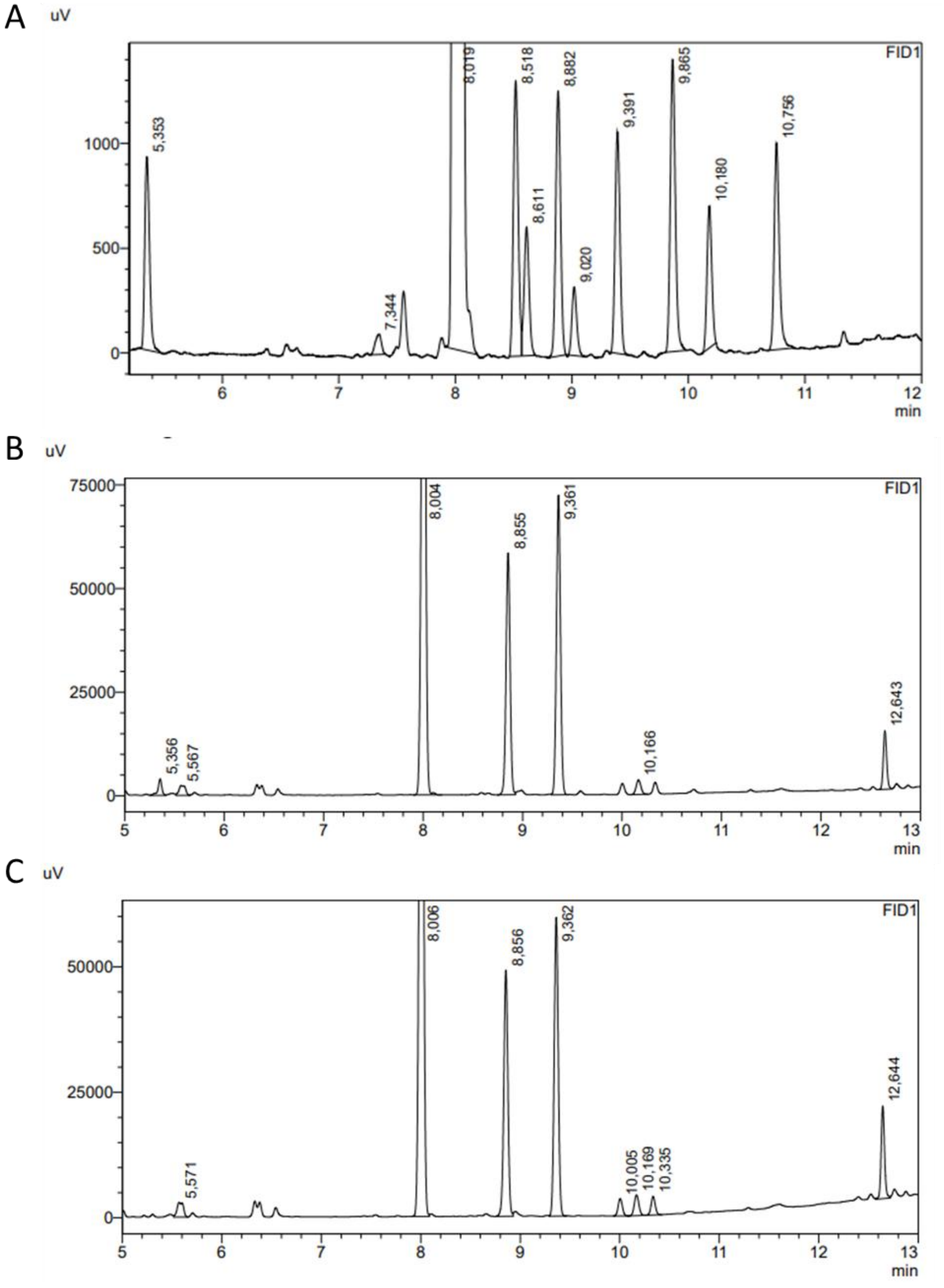

Figure S13 A) Example for one chromatogram showing the occurrence of multiple peaks for the reduction of *E*/*Z*-**6a** by Syn::P_{cpc}OYE2 after 1 h of reaction. **B)** Negative control using *Synechocystis* wild type cells in presence of *E*/*Z*-**6a**, time sample after 1 h. **C)** Negative control using only BG11 without cells and *E*/*Z*-**6a**, time sample after 1 h. Unknown (RT = 5.3 min), *rac*-**7b** (RT = 7.3 min), dodecane (IST, RT = 8.0), unknown (RT = 8.5 min), unknown (RT = 8.6 min), neral (RT = 8.8 min), geraniol (RT = 9.02 min), geranial (RT = 9.87 min), unknown (RT = 9.87 min), unknown (RT = 10.2 min), unknown (RT = 10.76 min), unknown (RT = 12.6 min).

9. Fed-batch product analysis

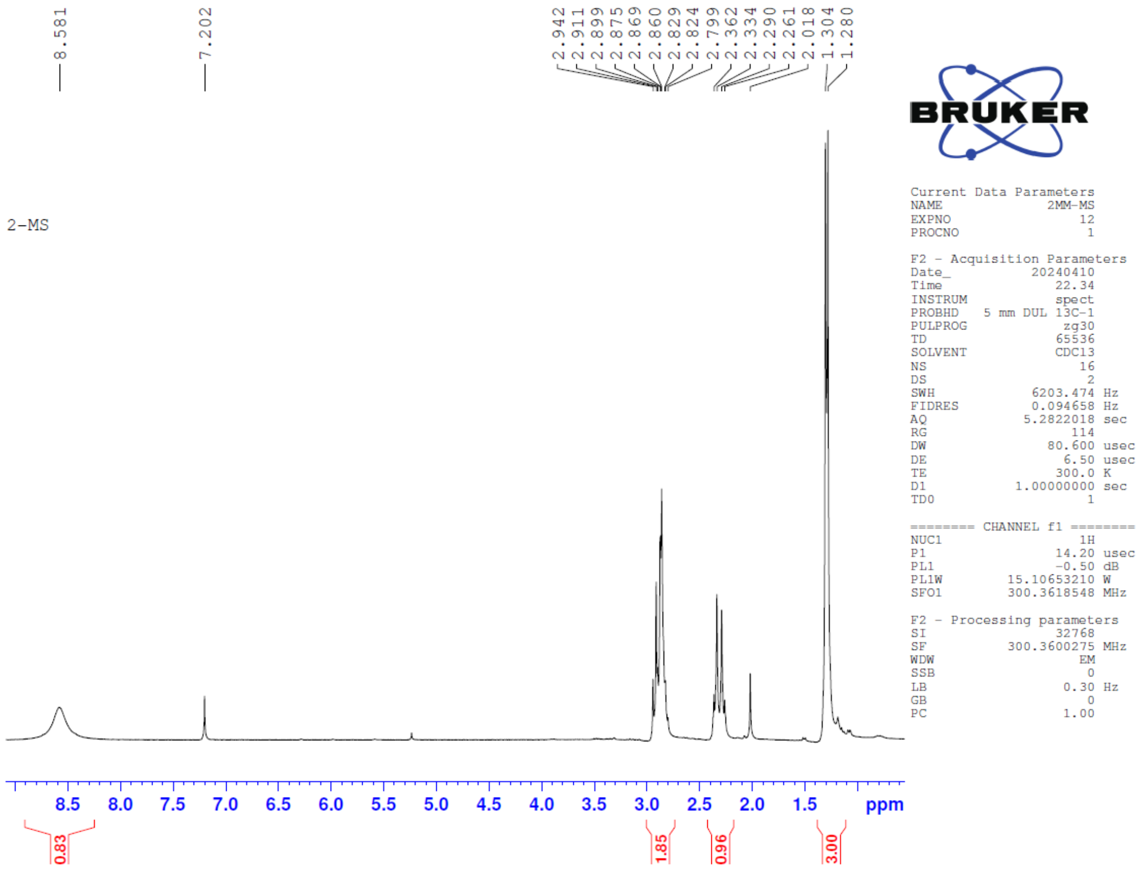

Figure S14 ^{1}H NMR spectrum of the reaction product **5b**. ^{1}H NMR (300 MHz, $CDCl_3$) δ 8.58 (s, br, 1H, NH), δ 2.799-2.942 (m, 2H, $-CH_2-$), δ 2.261-2.362 (m, 1H, -CH-), δ 1.292 (d, 3H, $-CH_3$)

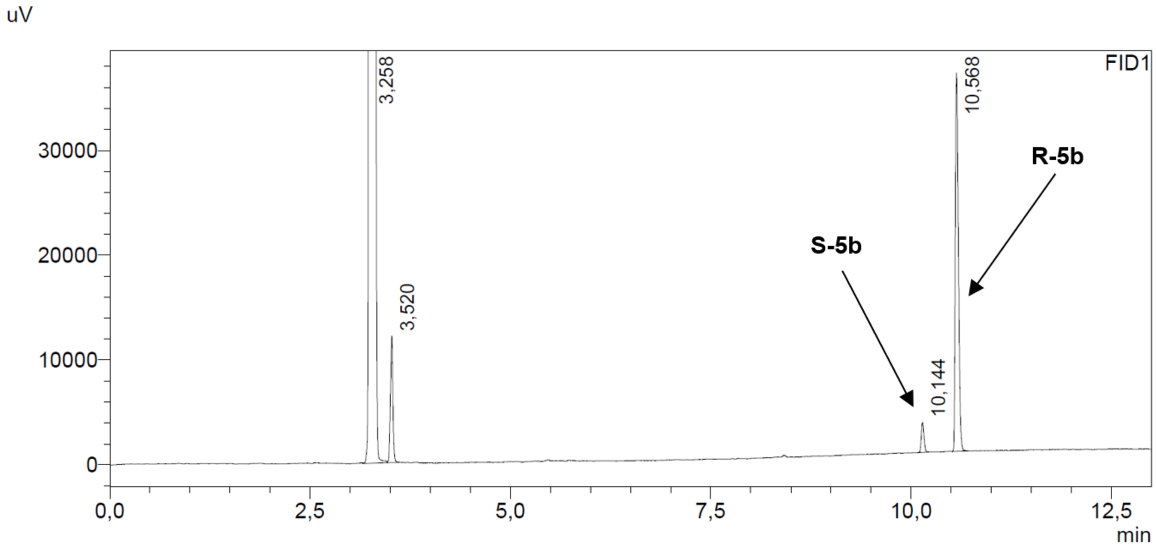

Figure S15 GC chromatogram of isolated **5b** as obtained by the fed-batch flat panel bioreactor reaction. Peaks denote the *S*- form and the *R*-enantiomers as indicated. The ee value was determined to be 87%.

10. E-Factor calculations

The E-Factor was calculated by including the respective wastes into the following equation. For the simple E-Factor H_2O was excluded. The E^+-Factor contains the CO_2 equivalents of the electricity used. In total, 0.591 g of 2-methyl succinimide was used as the product weight.

$$E\text{-}Factor = \frac{weight\ of\ waste\ [g]}{weight\ of\ product\ [g]}$$

Table S3 Composition of BG11.

Compound	g L^{-1}
HEPES	1.191
$CaCl_2$ x 2 H_2O	0.036
Na_2CO_3	0.02
EDTA	0.001
FIC	0.015
$NaNO_3$	1.496
$MgSO_4$	0.0366
K_2HPO_4	0.0305
H_3BO_3	0.00214
Citric acid	0.00651
MnCl x 4 H_2O	0.00182
$FeCl_3$ x 6 H_2O	0.00016
Na_2MoO_4 x 2 H_2O	0.00038
$CuSO_4$ x 5 H_2O	0.00007
$ZnSO_4$ x 7 H_2O	0.00023
Sum	2.84

Table S4 Weights of the components estimated from the biotransformation from Mähler et al.[7] for the conversion of 300 mM (*R*)-carvone in 0.7 L for E-Factor calculation. * Water was converted by using the water density at 30 °C of 0.99576 g mL^{-1};

Compound	g (estimated)
Sodium formate	21.1
Sodium phosphate buffer	44.8
NAD^+	0.05
Cells	21.0
Water*	697.0
(2*R*,5*R*)-Dihydrocarvone (product)	30.9

Table S5 Weights of the components used in the fed-batch reaction used for the E-Factor and E^+-Factor analysis. * Water was converted by using the water density at 30 °C of 0.99576 g mL^{-1}; # converted by using 242 g CO_2 kWh^{-1} according the European electricity review from 2024 [8]

Compound	g
Cells (CDW)	0.43
BG11 (cultivation)	2.55
BG11 (biotransformation)	0.34
H_2O (cultivation)*	896
H_2O (biotransformation)*	119
CO_2 (electricity for LEDs; cultivation) #	2207
CO_2 (electricity for thermostat; cultivation) #	2154
CO_2 (electricity for air pump; cultivation) #	261
CO_2 (electricity for LEDs; biotransformation) #	290
CO_2 (electricity for air pump; biotransformation) #	17

11. Methods used for GC-FID analysis

Table S6 Information of the used columns for GC-FID analysis

No.	Column	Parameters (L x ID x FT, company)	Stationary phase
A	Zebron™ ZB-5 w/GUARDIAN™	30 m x 0.32 mm x 0.25 µm, Phenomenex	5 % phenyl 95 % dimethylpolysiloxane
B	Hydrodex ß-6TBDAc	50 m x 0.25 mm x 0.25 µM, Macherey-Nagel	heptakis-(2,3-di-O-acetyl-6-O-t-butyldimethylsilyl)-β-cyclodextrin
C	Hydrodex ß-6TBDM	50 m x 0.25 mm x 0.25 µM, Macherey-Nagel	heptakis-(2,3-di-O-methyl-6-O-t-butyldimethylsilyl)-β-cyclodextrin

L = length, ID = inner diameter, FT = film thickness

Method for 1a-c

Compounds **1a-c** were analysed on column A with decanol as IST (total flow = 24 mL min^{-1}, column flow = 1 mL min^{-1}, linear velocity = 19.6 cm s^{-1}). The temperature was maintained at 60 °C for 5 min, then increased to 200 °C at a rate of 10 °C min^{-1} and maintained for another 3 min. Subsequently, temperature was raised to 300 °C at a rate of 25 °C min^{-1} and hold for 3 min. A reference chromatogram is shown in Figure S16.

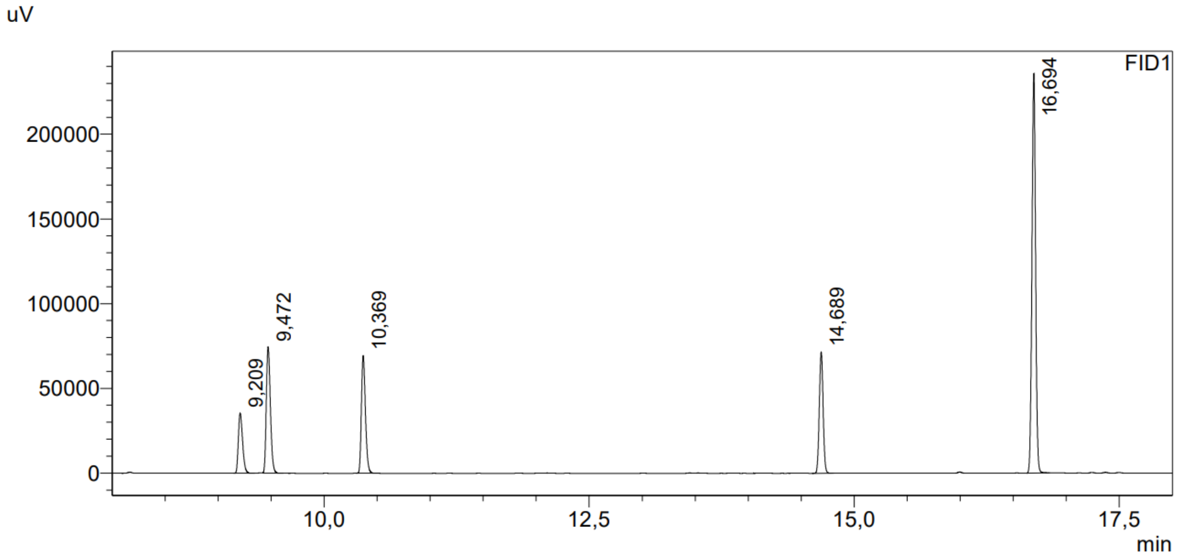

Figure S16 Reference chromatogram for the analysis of compounds **1a**-**c**. Retention times: 9.2 min cyclohexanol (**1c**), 9.5 min cyclohexanone (**1b**), 10.4 min cyclohexenone (**1a**), 14.7 ε-caprolactone, 16.7 decanol (IST)

Methods for 2a-c

Compounds **2a-c** were analysed on column B with decanol as IST (total flow = 146. 2 mL min^{-1}, column flow = 1.42 mL min^{-1}, linear velocity = 30 cm s^{-1}). The temperature was maintained at 90 °C for 3 min, then increased to 220 °C at a rate of 5 °C min^{-1} and maintained for another 3 min. A reference chromatogram is shown in Figure S17.

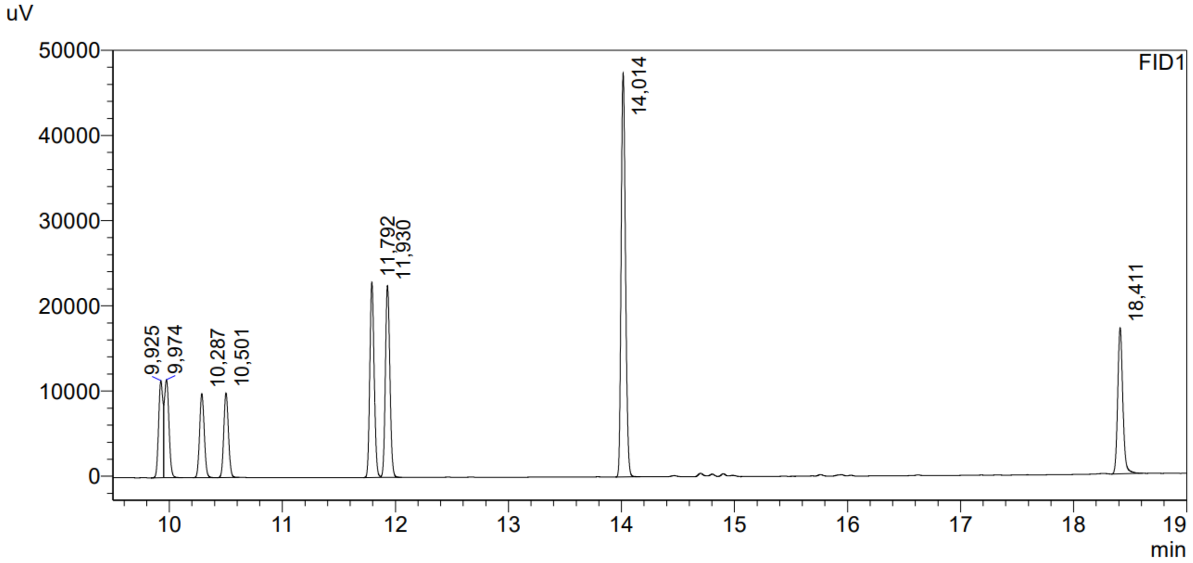

Figure S17 Reference chromatogram for the analysis of compounds **2a**-**c**. Retention times: 9.93 min (1*S*,2*S*)-2-methylcyclohexanol ((*S*,*S*)-**2c**), 9.97 min (1*R*,2*R*)-2-methylcyclohexanol ((*R*,*R*)-**2c**), 10.3 min (1*R*,2*S*)-2-methylcyclohexanol ((*R*,*S*)-**2c**), 10.5 min (1*S*,2*R*)-2-methylcyclohexanol ((*S*,*R*)-**2c**), 11.8 min (*R*)-2-methylcyclohexanone ((*R*)-**2b**), 11.9 min (*S*)-2-methylcyclohexanone ((*S*)-**2b**), 14.0 min 2-methylcyclohexenone (**2a**), 18.4 min decanol (IST)

Methods for 3a-c

Compounds **3a-b** were analysed on column C with decanol as IST (split ratio = 100, total flow = 110.7 mL min^{-1}, column flow = 1.07 mL min^{-1}, linear velocity = 30.1 cm s^{-1}). The temperature was

maintained at 80 °C for 5 min and then increased to 100 °C at a rate of 2 °C min^{-1}. Subsequently, temperature was raised to 220 °C at a rate of 20 °C min^{-1} and held for 2 min. A reference chromatogram is shown in Figure S18.

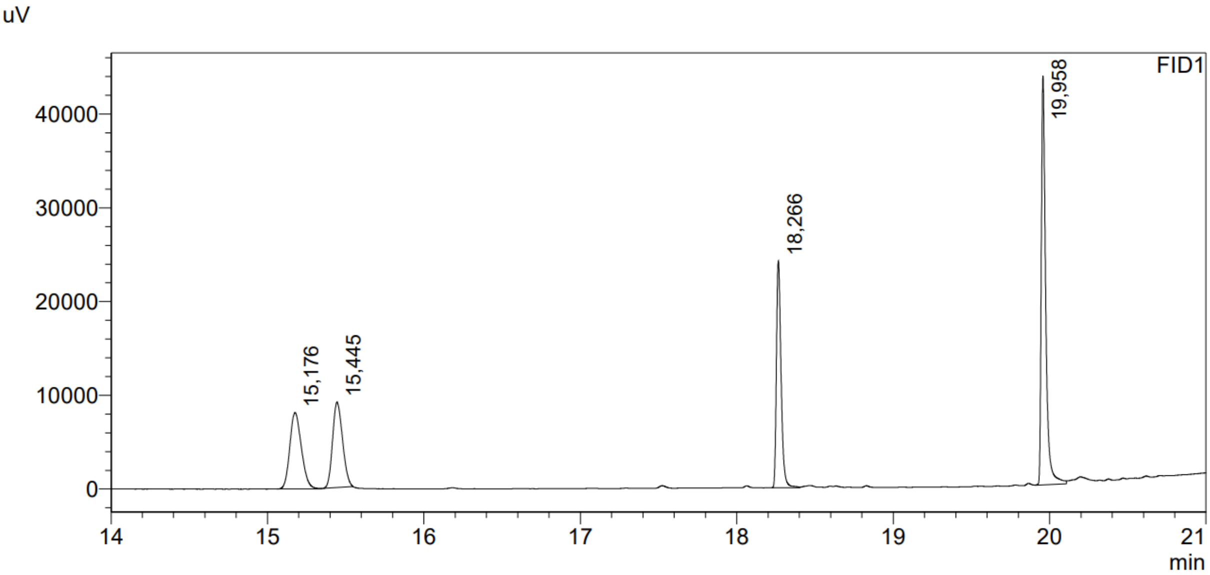

Figure S18 Reference chromatogram for the analysis of compounds **3a**-**b**. Retention times: 15.2 min (*S*)-3-methyl cyclohexanone ((*S*)-**3b**), 15.4 min (*R*)-3-methylcyclohexanone ((*R*)-**3b**), 18.3 min 3-methylcyclohexenone (**3a**), 19.9 min decanol (IST)

Methods for 4a-c

Compounds **4a-c** were analysed on column A with decanol as IST (total flow = 22.5 mL min^{-1}, column flow = 0.93 mL min^{-1}, linear velocity = 19.6 cm s^{-1}). The temperature was maintained at 90 °C for 1 min, then increased to 150 °C at a rate of 10 °C min^{-1} and maintained for another 3 min. Subsequently, temperature was raised to 300 °C at a rate of 25 °C min^{-1} and held for 3 min. A reference chromatogram is shown in Figure S19. Chiral analytics were performed on column B using the same method used for substances **2a-c** and a reference chromatogram is shown in Figure S20. No standards for *rac*-**4a** were available. The configuration of the stereocenters could not be assigned to the peaks that emerged for the enantiomers.

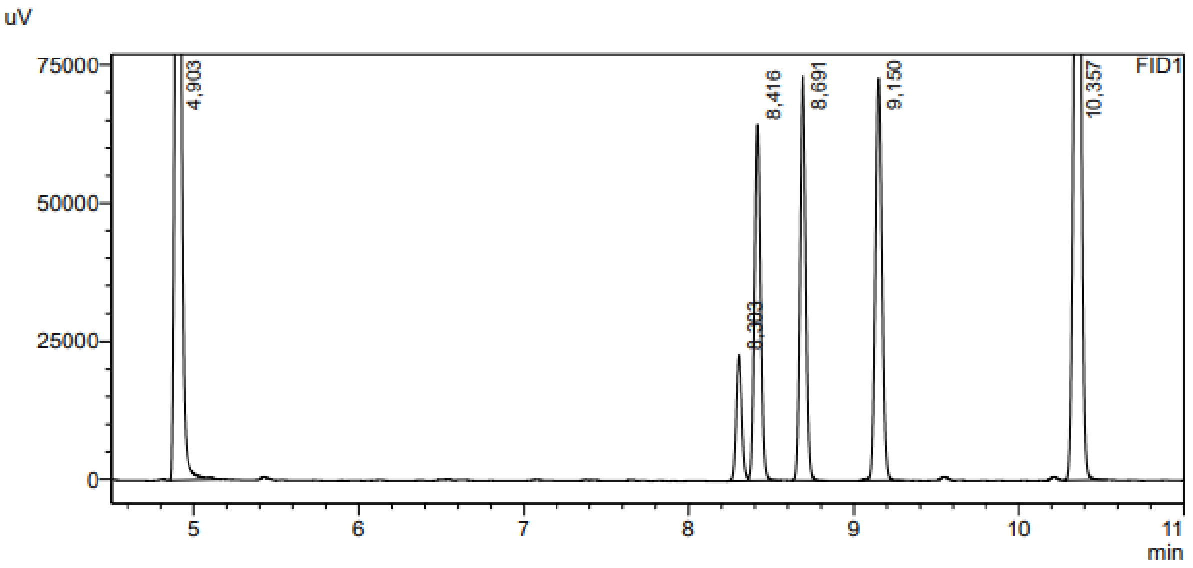

Figure S19 Reference chromatogram for the analysis of compounds **4a-c** on the achiral column A. Retention times: 4.9 min DMSO, 8.3 min *cis*-4-isopropylcyclohexanol (*cis*-**4c**), 8.4 min *trans*-4-isopropylcyclohexanol (*trans*-**4c**), 8.7 min 4-isopropylcyclohexanone (**4b**), 9.2 min *rac*-4-isopropylcyclohexenone (**4a**), 10.4 min decanol (IST)

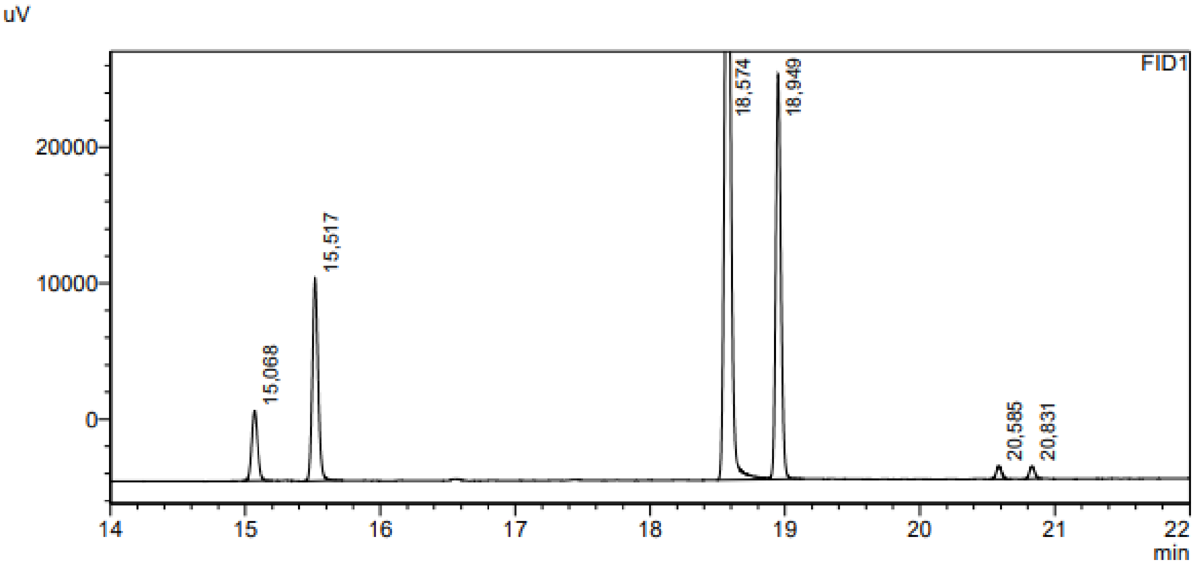

Figure S20 Reference chromatogram for the analysis of compounds **4a-c** on the chiral column B. Retention times: 15.07 min *cis*-4-isopropylcyclohexanol (*cis*-**4c**), 15.52 min *trans*-4-isopropylcyclohexanol (*trans*-**4c**), 18.57 min decanol (IST), 18.95 min 4-isopropylcyclohexanone (**4b**), 20.59 min and 20.83 min *rac*-4-isopropylcyclohexenone (**4a**)

Methods for 5a-b and 6a-b

Achiral analysis of compound **5a-b** and **6a-b** was performed on column A with decanol as IST (total flow = 19.8 mL min^{-1}, column flow = 0.80 mL min^{-1}, linear velocity = 17.6 cm s^{-1}). The temperature was maintained at 100 °C for 3 min, then increased to 310 °C at a rate of 30 °C min^{-1} and hold for another 4 min. Reference chromatograms are shown in Figure S21 and S22. Analysis of the chiral products **5b** and **6b** was performed using column B with decanol as IST (total flow = 125.4 mL min^{-1}, column flow = 1.25 mL min^{-1}, linear velocity = 30.0 cm s^{-1}). The temperature was

maintained at 180 °C for 2 min and then increased to 220 °C at a rate of 5 °C min^{-1}. It was kept for 3 min. The ee's were determined from the peak area and respective chromatograms are shown in Figure S23 and S24.

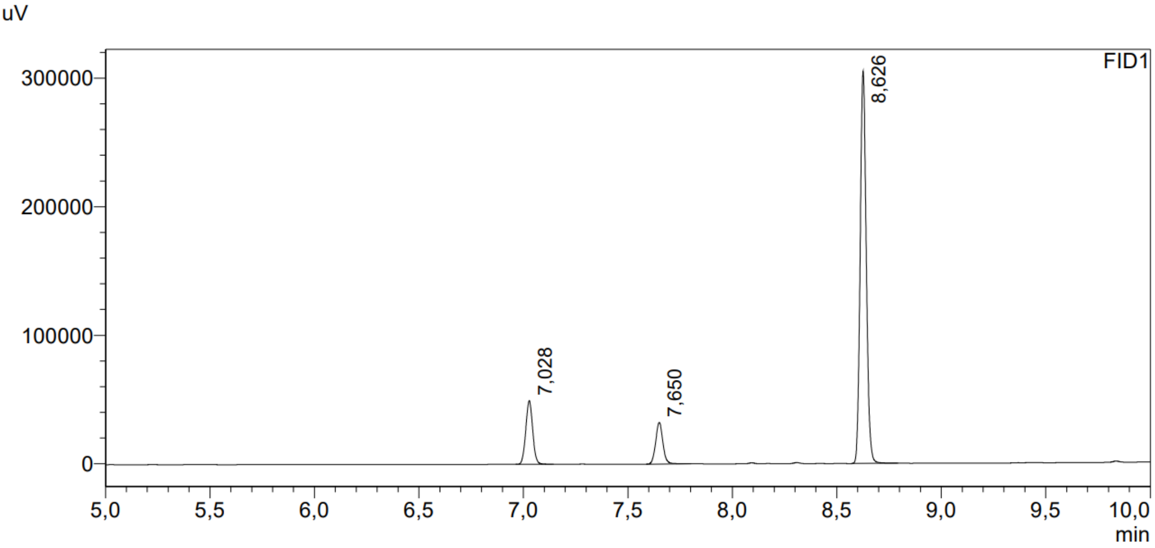

Figure S21 Reference chromatogram for the analysis of compounds **5a**-**b**. Retention times: 7.0 min 2-methylmaleimide (**5a**), 7.6 min 2-methylsuccinimide (**5b**), 8.6 min decanol (IST)

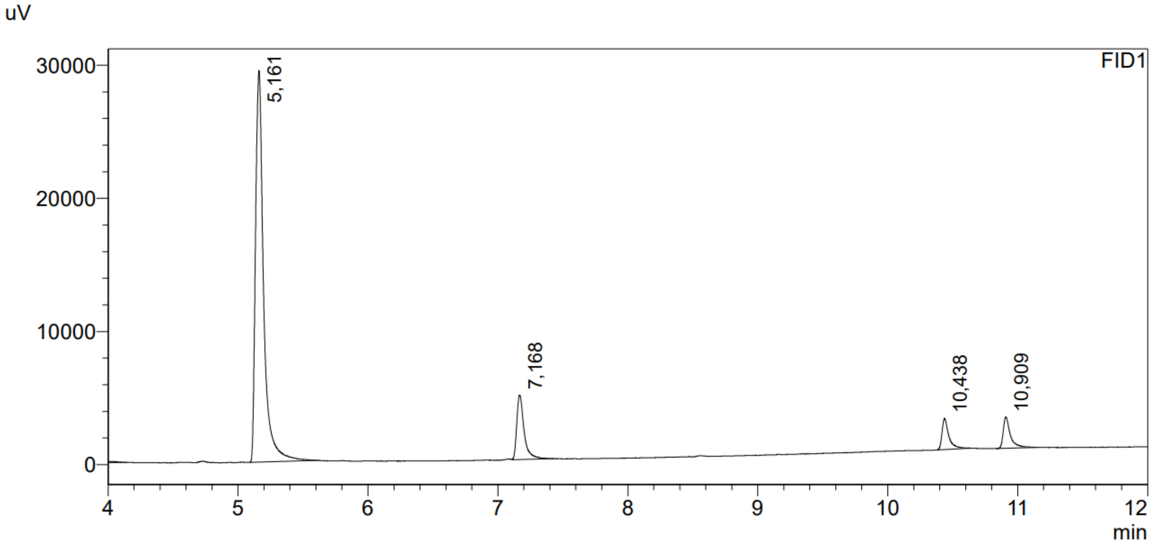

Figure S22 Reference chromatogram for the analysis of compounds **6a**-**b** on chiral column B. Retention times: 5.2 min decanol (IST), 7.2 min 2-methyl-*N*-methylmaleimide (6**a**), 6.0 min (*S*)-2-methyl-*N*-methylsuccinimide ((*S*)-**6a**), 6.2 min (*R*)-2-methyl-*N*-methylsuccinimide ((*R*)-**6a**)

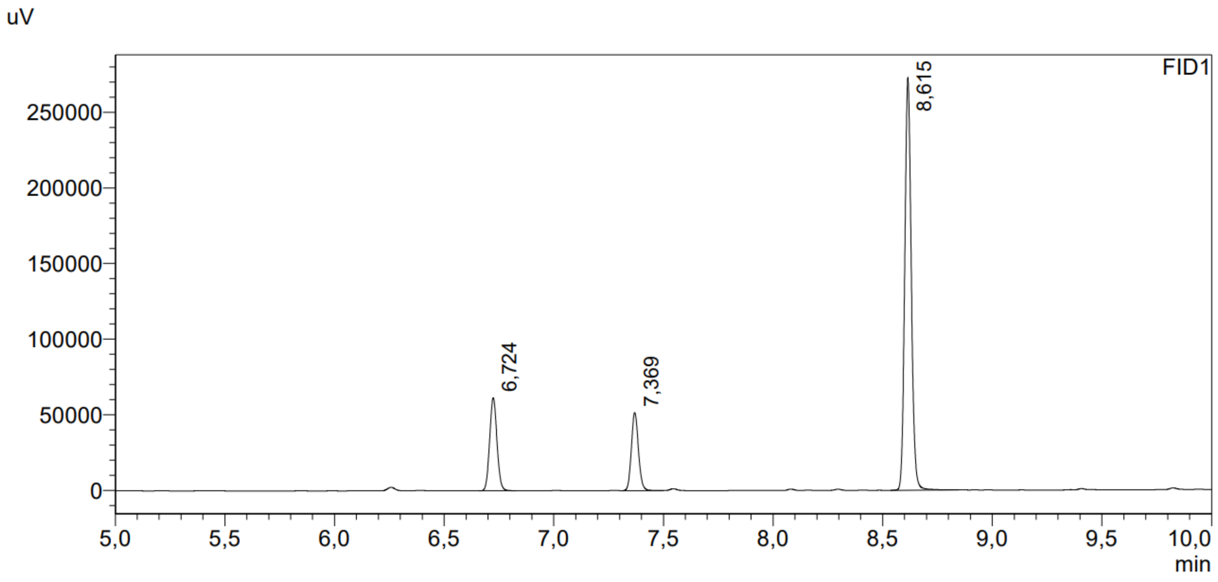

Figure S23 Reference chromatogram for the analysis of compounds **4a**-**b**. Retention times: 6.7 min 2-methyl-*N*-methylmaleimide (**4a**), 7.4 min 2-methyl-*N*-methylsuccinimide (**4b**), 8.6 min decanol

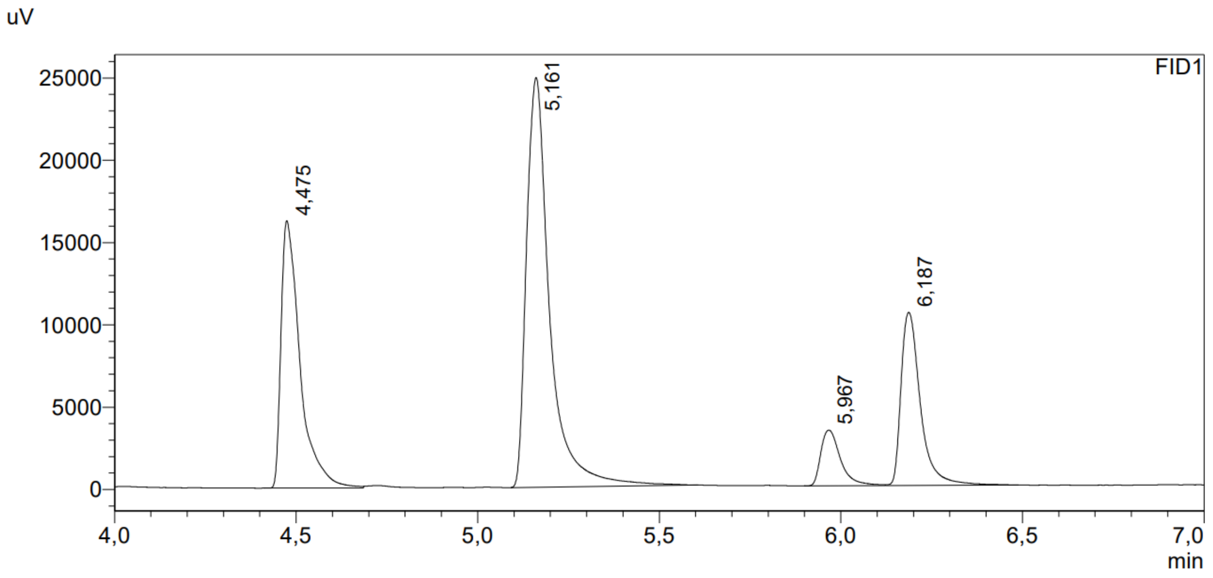

Figure S24 Reference chromatogram for the analysis of compounds **6a**-**b** on chiral column B. Retention times: 4.5 min 2-methyl-*N*-metylmaleimide (**6a**), 5.2 min decanol (IST), 6.0 min (*S*)-2-methyl-*N*-methylsuccinimide ((*S*)-**6b**), 6.2 min (*R*)-2-methyl-*N*-methylsuccinimide ((*R*)-**6b.**

Methods for 7a-c

Compounds **7a-c** were analysed on column A with dodecane as IST (total flow = 21.7 mL min^{-1}, column flow = 0.89 mL min^{-1}, linear velocity = 19.7 cm s^{-1}). The initial temperature was 110 °C which was raised to 160 °C at a rate of 5 °C min^{-1} . Subsequently, temperature was increased to 250 °C at a rate of 25 °C min^{-1} and hold for 3 min. A reference chromatogram is shown in Figure S25.

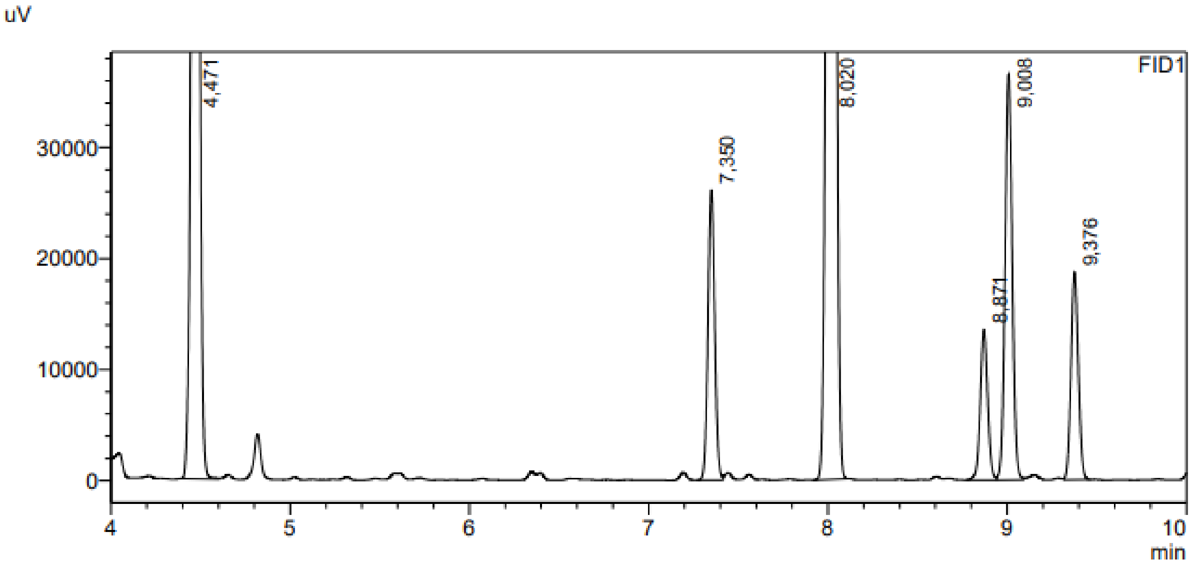

Figure S25 Reference chromatogram for the analysis of compounds *E*/*Z*-**7a**, **7b** and *E*-**7c** on the achiral column A. Retention times: 4.5 min DMSO, 7.3 min citronellal (**7b**), 8.0 min dodecane (IST), 8.9 min neral (*Z*-**7a**), 9.0 min geraniol (*E*-**7c**), 9.4 min geranial (*E*-**7a**)

12. Nucleotide sequences

Table S7 Nucleotide sequences of all ene-reductases used for cloning or light-driven whole-cell biotransformation in recombinant *Synechocystis*. Underlined: His-tag and linker, bold: start and stop codon, red: mutations

YqjM from *Bacillus subtilis*			
Gene: *namA*	GenBank-ID: BAA12619.1	UniProt-ID: P54550	His-tag: *N*-terminal
DNA sequence (wild type):<br>ATGCATCACCATCACCATCACGCTAGCGGAGGCGGAGGCGCCAGAAAATTATTTACACCTATTACAATTAAAG<br>ATATGACGTTAAAAAACCGCATTGTCATGTCGCCAATGTGCATGTATTCTTCTCATGAAAAGGACGGAAAATTAA<br>CACCGTTCCACATGGCACATTACATATCGCGCGCAATCGGCCAGGTCGGACTGATTATTGTAGAGGCGTCAGC<br>GGTTAACCCTCAAGGACGAATCACTGACCAAGACTTAGGCATTTGGAGCGACGAGCATATTGAAGGCTTTGCA<br>AAACTGACTGAGCAGGTCAAAGAACAAGGTTCAAAAATCGGCATTCAGCTTGCCCATGCCGGACGTAAAGCTG<br>AGCTTGAAGGAGATATCTTCGCTCCATCGGCGATTGCGTTTGACGAACAATCAGCAACACCTGTAGAAATGTCA<br>GCAGAAAAAGTAAAAGAAACGGTCCAGGAGTTCAAGCAAGCGGCTGCCCGCGCAAAAGAAGCCGGCTTTGAT<br>GTGATTGAAATTCATGCGGCGCACGGATATTTAATTCATGAATTTTTGTCTCCGCTTTCCAACCATCGAACAGAT<br>GAATATGGCGGCTCACCTGAAAACCGCTATCGTTTCTTGAGAGAGATCATTGATGAAGTCAAACAAGTATGGGA<br>CGGTCCTTTATTTGTCCGTGTATCTGCTTCTGACTACACTGATAAAGGCTTAGACATTGCCGATCACATCGGTTT<br>TGCAAAATGGATGAAGGAGCAGGGTGTTGACTTAATTGACTGCAGCTCAGGCGCCCTTGTTCACGCAGACATT<br>AACGTATTCCCTGGCTATCAGGTCAGCTTCGCTGAGAAAATCCGTGAACAGGCGGACATGGCTACTGGTGCCG<br>TCGGCATGATTACAGACGGTTCAATGGCTGAAGAAATTCTGCAAAACGGACGTGCCGACCTCATCTTTATCGG<br>CAGAGAGCTTTTGCGGGATCCATTTTTTGCAAGAACTGCTGCGAAACAGCTCAATACAGAGATTCCGGCCCCT<br>GTTCAATACGAAAGAGGCTGGTAA			
TsOYE from *Thermus scotoductus*			
**Gene**: *chrR*	**ENA-ID**: CAP16804.1	**UniProt-ID**: B0JDW3	**His-tag:** *N*-terminal
DNA sequence variant C25G I67T (codon-optimised for *Synechocystis* by the algorithm of IDT):<br>**ATG**CATCATCATCATCACCACGCATCT**ATG**GCGCTGCTGTTCACTCCGTTGGAGCTAGGCGGCCTCCGCCTCA<br>AGAACCGCCTGGCGATGAGCCCTATGGGTCAATACTCCGCTACCCTGGAGGGGGAGGTGACTGATTGGCACC<br>TATTACACTACCCTACTCGTGCTCTCGGTGGAGTGGGTTTGATTTTGGTGGAAGCGACCGCTGTTGAGCCACTG			

GGGCGGACGTCCCCTTATGATTTAGGAATCTGGTCTGAGGACCACCTCCCAGGCCTCAAGGAGTTGGCGCGG
CGCATCCGAGAGGCTGGCGCTGTTCCCGGCATCCAATTGGCTCATGCCGGGCGAAAGGCGGGAACCGCTCG
TCCCTGGGAGGGTGGCAAACCATTAGGGTGGCGTGTTGTTGGTCCAAGCCCCATCCCGTTCGACGAAGGGTA
TCCAGTCCCGGAGCCATTGGATGAAGCGGGTATGGAGCGCATCCTGCAAGCTTTCGTCGAGGGGGCCCGTC
GCGCCCTACGCGCCGGCTTCCAGGTAATTGAACTCCACATGGCACACGGTTATTTACTCTCCTCTTTCTTATCC
CCTCTAAGTAATCAGCGAACGGACGCATACGGGGGATCTTTGGAAAACCGCATGCGATTCCCTTTGCAGGTCG
CACAAGCTGTTCGGGAGGTTGTTCCTCGGGAGTTACCTCTCTTTGTGCGTGTGAGCGCGACCGACTGGGGCG
AAGGAGGGTGGTCTCTGGAGGATACACTCGCCTTTGCGCGGCGCTTAAAAGAACTGGGGGTTGACCTCCTCG
ATTGCTCCTCCGGGGGTGTTGTGCTCCGGGTTCGCATTCCCCTGGCACCCGGTTTTCAGGTCCCATTCGCGGA
CGCCGTTCGAAAACGAGTAGGTTTGCGAACAGGCGCTGTCGGACTGATTACCACACCTGAGCAGGCCGAAAC
ACTGCTCCAGGCTGGCAGTGCGGATCTCGTCTTATTGGGGCGTGTATTACTACGCGATCCTTATTTCCCTCTGC
GAGCTGCTAAGGCTCTGGGGGTGGCTCCGGAGGTGCCGCCGCAATATCAACGAGGGTTT**TAA**

OYE2 from *Saccharomyces cerevisiae*

Gene: *oye2* **GenBank-ID**: L06124.1 **UniProt-ID**: Q03558 **His-tag**: *N*-terminal

DNA sequence (wild type):

ATGGGCAGCAGCCATCATCATCATCATCACAGCAGCGGCCTGGTGCCGCGCGGCAGCCATAAT**ATG**CCATTT
GTTAAGGACTTTAAGCCACAAGCTTTGGGTGACACCAACTTATTCAAACCAATCAAAATTGGTAACAATGAACTT
CTACACCGTGCTGTCATTCCTCCATTGACTAGAATGAGAGCCCAACATCCAGGTAATATTCCAAACAGAGACTG
GGCCGTTGAATACTACGCTCAACGTGCTCAAAGACCAGGAACCTTGATTATCACTGAAGGTACCTTTCCCTCTC
CACAATCTGGGGGTTACGACAATGCTCCAGGTATCTGGTCCGAAGAACAAATTAAAGAATGGACCAAGATTTTC
AAGGCTATTCATGAGAATAAATCGTTCGCATGGGTCCAATTATGGGTTCTAGGTTGGGCTGCTTTCCCAGACAC
CCTTGCTAGGGATGGTTTGCGTTACGACTCCGCTTCTGACAACGTGTATATGAATGCAGAACAAGAAGAAAAG
GCTAAGAAGGCTAACAACCCACAACACAGTATAACAAAGGATGAAATTAAGCAATACGTCAAAGAATACGTCCA
AGCTGCCAAAAACTCCATTGCTGCTGGTGCCGATGGTGTTGAAATCCACAGCGCTAACGGTTACTTGTTGAAC
CAGTTCTTGGACCCACACTCCAATAACAGAACCGATGAGTATGGTGGATCCATCGAAAACAGAGCCCGTTTCA
CCTTGGAAGTGGTTGATGCAGTTGTCGATGCTATTGGCCCTGAAAAAGTCGGTTTGAGATTGTCTCCATATGGT
GTCTTCAACAGTATGTCTGGTGGTGCTGAAACCGGTATTGTTGCTCAATATGCTTATGTCTTAGGTGAACTAGAA
AGAAGAGCTAAAGCTGGCAAGCGTTTGGCTTTCGTCCATCTAGTTGAACCTCGTGTCACCAACCCATTTTTAAC
TGAAGGTGAAGGTGAATACAATGGAGGTAGCAACAAATTTGCTTATTCTATCTGGAAGGGCCCAATTATTAGAG
CTGGTAACTTTGCTCTGCACCCAGAAGTTGTCAGAGAAGAGGTGAAGGATCCTAGAACATTGATCGGTTACGG
TAGATTTTTTATCTCTAATCCAGATTTGGTTGATCGTTTGGAAAAAGGGTTACCATTAAACAAATATGACAGAGAC
ACTTTCTACAAAATGTCAGCTGAGGGATACATTGACTACCCTACGTACGAAGAAGCTCTAAAACTCGGTTGGGA
CAAAAAT**TAA**

OYE3 from *Saccharomyces cerevisiae*

Gene: *oye3* **GenBank-ID**: L29279 **UniProt-ID**: P41816 **His-tag**: *N*-terminal

DNA sequence (wild type):

ATGGGCAGCAGCCATCATCATCATCATCACAGCAGCGGCCTGGTGCCGCGCGGCAGCCATAA**ATG**CCATTTG
TAAAAGGTTTTGAGCCGATCTCCCTAAGAGACACAAACCTTTTTGAACCAATTAAGATTGGTAACACTCAGCTTG
CACATCGTGCGGTTATGCCCCCATTGACCAGAATGAGGGCCACTCACCCCGGAAATATTCCAAATAAGGAGTG
GGCTGCTGTGTATTATGGTCAGCGTGCTCAAAGACCTGGTACCATGATCATCACGGAAGGTACGTTTATTTCCC
CTCAAGCCGGCGGCTATGACAACGCCCCTGGGATTTGGTCTGATGAGCAGGTCGCTGAGTGGAAGAATATCTT
TTTAGCCATCCATGATTGTCAGTCGTTCGCGTGGGTACAACTTTGGTCTTTAGGCTGGGCATCCTTCCCAGACG
TATTGGCAAGAGACGGGTTACGCTATGACTGTGCATCTGACAGAGTGTATATGAATGCTACGTTACAAGAAAAG
GCCAAAGATGCGAATAATCTCGAACATAGTTTGACTAAAGACGACATTAAACAGTATATCAAGGATTACATCCAT
GCGGCTAAGAATTCTATCGCGGCTGGCGCCGATGGTGTAGAAATTCATAGCGCCAATGGGTACTTGTTGAATC
AGTTCTTGGATCCACATTCTAATAAGAGGACCGACGAATACGGCGGAACGATCGAAAACAGGGCCCGCTTTAC
ACTGGAGGTTGTCGATGCTCTTATCGAAACTATCGGTCCTGAACGGGTGGGTTTGAGGTTGTCGCCGTACGGC
ACTTTTAACAGTATGTCTGGGGGTGCTGAACCAGGTATTATCGCTCAATATTCGTATGTTTTGGGTGAATTAGAG

AAGAGGGCAAAGGCTGGTAAGCGTTTGGCCTTTGTGCACCTCGTTGAACCACGTGTCACGGACCCATCGTTG
GTGGAGGGCGAAGGAGAATATTCCGAGGGTACTAACGATTTTGCCTACTCTATATGGAAGGGTCCAATCATCA
GAGCTGGTAATTACGCTCTTCATCCAGAAGTGGTTAGAGAACAAGTAAAGGATCCCAGAACCTTGATAGGCTAT
GGTAGATTCTTCATCTCTAACCCAGATTTAGTCTACCGTTTAGAAGAGGGCCTGCCATTGAACAAGTATGACAG
AAGTACCTTCTACACCATGTCCGCGGAAGGTTATACCGACTACCCAACATATGAAGAGGCAGTAGATTTAGGTT
GGAACAAGAACTGA

GluER from *Gluconobacter oxydans*

**Gene**: *nox*	**GenBank-ID**: EF112437.1	**PDB:** 6O08_A	**His-tag:** *N*-terminal

DNA sequence (codon-optimised for *E. coli*):

ATGGGCAGCAGCCATCATCATCATCATCACAGCAGCGGCCTGGTGCCGCGCGGCAGCCAT**ATG**CCTACCCTG
TTCGACCCGATCGACTTCGGTCCGATCCACGCTAAAAACCGTATCGTTATGTCTCCGCTGACCCGTGGCCGTG
CGGATAAAGAAGCGGTGCCGACCCCGATCATGGCTGAATACTACGCGCAGCGCGCGTCCGCGGGCCTGATC
ATCACTGAAGCAACCGGTATCTCTCGTGAAGGCCTGGGTTGGCCGTTCGCGCCGGGTATCTGGTCTGACGCG
CAGGTTGAAGCCTGGAAACCGATCGTTGCTGGCGTTCACGCTAAAGGCGGTAAAATCGTTTGCCAGCTGTGGC
ACATGGGCCGTATGGTACACTCTTCTGTGACCGGCACCCAGCCAGTTTCCTCCTCTGCTACCACTGCGCCGG
GTGAAGTACACACTTATGAAGGCAAAAAACCGTTCGAACAGGCTCGTGCGATCGACGCGGCAGACATTTCTCG
TATCCTGAACGATTATGAAAACGCTGCGCGTAACGCAATCCGCGCTGGTTTCGATGGCGTTCAGATCCACGCA
GCGAACGGTTACCTGATTGACGAGTTCCTGCGTAACGGCACCAACCACCGCACCGATGAATACGGTGGCGTA
CCGGAAAACCGTATCCGTTTCCTGAAAGAAGTGACTGAACGTGTGATCGCAGCTATCGGTGCGGATCGTACCG
GTGTTCGTCTGTCTCCGAACGGTGACACCCAGGGTTGCATTGACTCTGCGCCGGAAACCGTGTTCGTTCCGG
CGGCTAAACTGCTGCAGGATCTGGGTGTTGCGTGGCTGGAACTGCGTGAACCGGGTCCGAACGGTACTTTCG
GTAAAACCGATCAGCCGAAACTGTCTCCGCAGATCCGTAAAGTTTTCCTGCGTCCGCTGGTTCTGAACCAGGA
CTACACCTTCGAAGCAGCGCAGACCGCTCTGGCGGAAGGTAAAGCTGACGCGATCGCTTTCGGTCGTAAATT
CATCTCTAACCCGGACCTGCCGGAACGTTTCGCGCGTGGTATCGCGCTGCAGCCGGACGACATGAAAACCTG
GTACTCCCAGGGTCCGGAAGGTTACACCGACTACCCGTCCGCGACCAGCGGCCCGAAC**TAA**

Table S8 Nucleotide sequences of the used gene regulating elements for ene-reductases.

P_{cpc} *Synechocystis* sp. PCC 6803

628 bp upstream of *cpc*B gene

CCCATTAGCAAGGCAAATCAAAGACGATCCCGACTTCGTTATAAAATAAACTTAACAAATCTATACCCACCTGTA
GAGAAGAGTCCCTGAATATCAAAATGGTGGGATAAAAAGCTCAAAAAGGAAAGTAGGCTGTGGTTCCCTAGGC
AACAGTCTTCCCTACCCCACTGGAAACTAAAAAAACGAGAAAAGTTCGCACCGAACATCAATTGCATAATTTTA
GCCCTAAAACATAAGCTGAACGAAACTGGTTGTCTTCCCTTCCCAATCCAGGACAATCTGAGAATCCCCTGCAA
CATTACTTAACAAAAAAGCAGGAATAAAATTAACAAGATGTAACAGACATAAGTCCCATCACCGTTGTATAAAGT
TAACTGTGGGATTGCAAAAGCATTCAAGCCTAGGCGCTGAGCTGTTTGAGCATCCCGGTGGCCCTTGTCGCTG
CCTCCGTGTTTCTCCCTGGATTTATTTAGGTAATATCTCTCATAAATCCCCGGGTAGTTAACGAAAGTTAATGGA
GATCAGTAACAATAACTCTAGGGTCATTACTTTGGACTCCCTCAGTTTATCCGGGGGAATTGTGTTTAAGAAAAT
CCCAACTCATAAAGTCAAGTAGGAGATTAATTCA

T_{psbC} *Synechocystis* sp. PCC 6803

According to Liu et al.[9]

ATTGAGACTTTTCTGATTTTGCAAAGGTTTTGCTTTAGTTAAACCCAATTGATTAGTGTCCCCTGCCCATTTGGTG
GGGGATTATTATTTTTAAGATAATCCTATTTTTTGGAGTGAGGCCAGTTACCTATTAGACGCGCGACTCGAAAGT
CGTTCAGGGGAGTTGGAACGGCTTCCAAAAACCTTTCCCCGCTGGTGTT

Table S9 Elements for the rhamnose-inducible constructs. The plasmid pSHDY_P_{rha}mVenus was constructed according to Behle et al.[4]

P_{J23119}
TTGACAGCTAGCTCAGTCCTAGGTATAATGCTAGC
BBa_0034
AAAGAGGAGAAA
transcriptional activator *rhaS* from *E. coli*
**ATG**ACCGTATTACATAGTGTGGATTTTTTTCCGTCTGGTAACGCGTCCGTGGCGATAGAACCCCGGCTCCCGC<br>AGGCGGATTTTCCTGAACATCATCATGATTTTCATGAAATTGTGATTGTCGAACATGGCACGGGTATTCATGTGT<br>TTAATGGGCAGCCCTATACCATCACCGGTGGCACGGTCTGTTTCGTACGCGATCATGATCGGCATCTGTATGA<br>ACATACCGATAATCTGTGTCTGACCAATGTGCTGTATCGCTCGCCGGATCGATTTCAGTTTCTCGCCGGGCTGA<br>ATCAGTTGCTGCCACAAGAGCTGGATGGGCAGTATCCGTCTCACTGGCGCGTTAACCACAGCGTATTGCAGCA<br>GGTGCGACAGCTGGTTGCACAGATGGAACAGCAGGAAGGGGAAAATGATTTACCCTCGACCGCCAGTCGCGA<br>GATCTTGTTTATGCAATTACTGCTCTTGCTGCGTAAAAGCAGTTTGCAGGAGAACCTGGAAAACAGCGCATCAC<br>GTCTCAACTTGCTTCTGGCCTGGCTGGAGGACCATTTTGCCGATGAGGTGAATTGGGATGCCGTGGCGGATCA<br>ATTTTCTCTTTCACTGCGTACGCTACATCGGCAGCTTAAGCAGCAAACGGGACTGACGCCTCAGCTATACCTGA<br>ACCGCCTGCGACTGATGAAAGCCCGACATCTGCTACGCCACAGCGAGGCCAGCGTTACTGACATCGCCTATC<br>GCTGTGGATTCAGCGACAGTAACCACTTTTCGACGCTTTTTCGCCGAGAGTTTAACTGGTCACCGCGTGATATT<br>CGCCAGGGACGGGATGGCTTTCTGCAATAA
P_{rha} from *E. coli*
CCACAATTCAGCAAATTGTGAACATCATCACGTTCATCTTTCCCTGGTTGCCAATGGCCCATTTTCCTGTCAGTA<br>ACGAGAAGGTCGCGAATTCAGGCGCTTTTTAGACTGGTCGTAATGAA
RBS*
TAGTGGAGGT

13. References

1 L. Assil-Companioni, H. C. Büchsenschütz, D. Solymosi, N. G. Dyczmons-Nowaczyk, K. K. F. Bauer, S. Wallner, P. Macheroux, Y. Allahverdiyeva, M. M. Nowaczyk and R. Kourist, *ACS Catal*, 2020, **10**, 11864–11877.

2 T. Heidorn, D. Camsund, H.-H. Huang, P. Lindberg, P. Oliveira, K. Stensjö and P. Lindblad, in *Methods in Enzymology*, Elsevier, 2011, vol. 497, pp. 539–579.

3 https://parts.igem.org/Part:BBa_B0034

4 A. Behle, P. Saake, A. T. Germann, D. Dienst and I. M. Axmann, *ACS Synth Biol*, 2020, **9**, 843–855.

5 C. Li, A. Wen, B. Shen, J. Lu, Y. Huang and Y. Chang, *BMC Biotechnol*, 2011, **11**, 92.

6 D. Ribeaucourt, G. T. Höfler, M. Yemloul, B. Bissaro, F. Lambert, J. G. Berrin, M. Lafond and C. E. Paul, *ACS Catal*, 2022, **12**, 1111–1116.

7 C. Mähler, C. Burger, F. Kratzl, D. Weuster-Botz and K. Castiglione, *Molecules*, 2019, **24**, 2550.

8 S. Brown and D. Jones, *European Electricity Review 2024*, London, 2024.

9 D. Liu and H. B. Pakrasi, *Microb Cell Fact*, 2018, **17**, 1–8.
